# Supplementary material for: Examining the Role of a Chromosomal Inversion in Accumulating Adaptive and Barrier Loci in a Cold-adapted Insect Species
Source: Genome Biol Evol. 2026 Apr 17;18(5):evag101. doi: 10.1093/gbe/evag101 (PMC13171183; doi:10.1093/gbe/evag101)
Supplement: evag101_Supplementary_Data [file evag101_supplementary_data.zip › Dmontana_inversion_polymorphism_Supplementary_material_revised3_Poikela_etal.pdf]

Examining the role of a chromosomal inversion in accumulating adaptive and barrier loci in a cold-adapted insect species

Poikela N., Hoikkala V., Ritchie M. G. & Kankare M.

Table of Contents

Supplementary Tables......2
Table S1 2
Table S2 3
Table S3 4
Table S4 4
Table S5 4
Table S6 5
Table S7 6
Table S8 7
Table S9 9
Table S10 9
Table S11 10
Table S12 10
Table S13 11
Table S14 12
Table S15 13
Table S16 14
Table S17 15
Table S18 16
Table S19 18
Table S20 18
Table S21 18

Supplementary Figures......19
Figure S1 19
Figure S2 22
Figure S3 24
Figure S4 27
Figure S5 30
Figure S6 31
Figure S7 33
Figure S8 36
Figure S9 38
Figure S10 41
Figure S11 43
Figure S12 44
Figure S13 45
Figure S14. 46
Figure S15. 47
Figure S16 47
Figure S17 48

Supplementary references 48

Supplementary Tables

Table S1. Summary of the *D. montana* data used in the study. Table shows sampling locations, coordinates and years, the data used in cold experiments, the isofemale strain ID sequenced with PacBio, the number of wild-caught individuals sequenced with Illumina, and the availability of poolseq data for each population. Details of the PacBio and Illumina samples are given in Tables S7-S9.

Sampling location	Sampling year	Data used in cold experiments	Strain used in PacBio sequencing	Number of wild-caught females sequenced with Illumina	Illumina poolseq (49-50 single wild-caught females/males per pool)
**Western coast of North America**					
Seward, Alaska, USA<br>60°10'N; 149°27'W<br>Altitude 35 m	2013	mass-bred population	monSE13F37	11	yes
Terrace, British Columbia, Canada<br>54°27'N; 128°34'W<br>Altitude 217 m	2014	mass-bred population	monTER14F11	11	yes
Vancouver, British Columbia, Cana<br>49°15'N; 123°10'W<br>Altitude 4 m	2014	mass-bred population	-	10	no
Ashford, Washington, Usa<br>46°45'N; 121°57'W<br>Altitude 573 m	2013	mass-bred population	monASH13F9	10	yes
Azalea, Oregon, USA<br>42°48'N; 123°13'W<br>Altitude 498 m	2010	-	monAZA2	3	no
**Rocky Mountains of North America**					
McBride, British Columbia, Canadi<br>53°18'N; 120°9'W<br>Altitude 720 m	2014	mass-bred population	monMB14F1	9	no
Jackson, Wyoming, USA<br>43°26'N; 110°50'W<br>Altitude 1 857 m	2013	-	monJX13F48	3	no
Crested Butte, Colorado, USA<br>39°49'N; 107°04'W<br>Altitude 2960 m	2009	mass-bred population	mon34CC5	10	yes
**Fennoscandia**					
Storslett, Norway<br>69°36'N; 21°19'E<br>Altitude 2 m	2020	mass-bred population	monSTO20F2	10	no
Raattama, Finland<br>68°01'N; 24°08'E<br>Altitude 343 m	2020	-	monRAA20F1	3	no
Oulanka, Finland<br>66°22'N; 29°20'E<br>Altitude 341 m	2013	5 isofemale strains: OU21F1, OU21F14, OU21F15, OU21F30	monOU13F149	11	yes
Korpilahti, Finland<br>62°00' N; 25°34'E<br>Altitude 85 m	2013	mass-bred population	-	7	yes
**Kamchatka**					
Kamchatka peninsula, Russia<br>56°12'N; 161°59'E<br>Altitude 21 m	2013	-	monKR1309	3	no

Table S2. Latitude, longitude, altitude and 19 bioclimatic variables and their description for each fly sampling site. The bioclimatic variables were extracted from WorldClim database v2.1 with 2.5 min spatial resolution using geographic coordinates (latitudes and longitudes) of each fly collecting site (Fick & Hijmans 2017). The darker (greener) the colour, the greater the value. NA = North America.

Geographic region	Population	Latitude	Longitude	Altitude	bio1	bio2	bio3	bio4	bio5	bio6	bio7	bio8	bio9	bio10	bio11	bio12	bio13	bio14	bio15	bio16	bio17	bio18	bio19
Western coast (NA)	Seward	60.17	-149.45	35	2.26	6.77	28.76	639.63	15.62	-7.91	23.54	2.06	8.53	10.68	-4.66	1266	197	54	44.92	505	178	213	342
	Terrace	54.45	-128.57	217	6.41	7.72	27.57	730.96	21.38	-6.63	28.01	1.75	13.43	15.32	-2.71	1379	210	50	51.67	578	157	170	487
	Vancouver	49.25	-123.17	4	10.28	6.76	31.70	533.84	22.28	0.95	21.34	4.59	16.87	17.01	4.04	1440	225	45	51.90	620	154	157	536
	Ashford	46.75	-121.95	573	7.55	10.18	41.90	514.42	22.52	-1.77	24.29	2.13	14.00	14.22	1.86	2025	309	43	57.67	917	178	189	832
	Azalea	42.80	-123.22	498	11.05	13.74	48.03	555.79	28.90	0.30	28.60	5.03	18.11	18.20	4.80	1145	196	13	70.09	559	62	71	507
Rocky Mountains (NA)	McBride	53.30	-120.16	720	4.07	11.31	32.24	850.10	22.13	-12.94	35.07	4.04	0.10	14.23	-6.66	706	75	39	21.11	210	127	201	163
	Jackson	43.43	-110.83	1857	3.65	16.12	38.11	943.61	27.08	-15.21	42.29	8.40	-1.65	15.16	-8.27	439	50	27	20.40	128	90	108	115
	Crested Butte	39.82	-107.07	2960	3.58	17.14	42.68	848.91	25.25	-14.92	40.16	13.79	-6.56	14.09	-6.56	430	45	29	13.34	127	97	122	97
Fennoscandia	Storslett	69.60	21.32	95	1.42	5.85	22.07	757.24	15.73	-10.80	26.53	10.34	-4.12	11.32	-7.17	482	62	26	28.02	163	83	153	110
	Raattama	68.02	24.13	343	-1.74	9.03	24.14	995.77	17.76	-19.64	37.41	11.12	-8.01	11.12	-13.60	523	85	26	44.73	221	79	221	87
	Oulanka	66.37	29.33	341	-0.81	9.74	24.54	1037.88	19.78	-19.91	39.69	12.43	-7.76	12.43	-13.48	571	76	29	31.99	206	92	206	101
	Korpilahti	62.00	25.57	85	3.51	7.58	23.02	899.21	21.16	-11.75	32.91	13.17	-3.19	14.98	-7.34	614	86	29	34.21	225	100	220	116
Kamchatka	Kamchatka	56.20	161.98	21	-0.46	7.27	22.74	928.28	15.79	-16.18	31.97	-9.46	2.60	10.79	-11.69	691	77	35	26.39	218	110	171	211

bio1	Annual mean temperature
bio2	Mean diurnal range (mean of monthly (max - min temperature))
bio3	Isothermality (bio2/bio7*100)
bio4	Temperature seasonality (standard deviation * 100)
bio5	Max temperature of the warmest month
bio6	Min temperature of the coldest month
bio7	Annual temperature range (bio5-bio6)
bio8	Mean temperature of the wettest quarter
bio9	Mean temperature of the driest quarter
bio10	Mean temperature of the warmest quarter
bio11	Mean temperature of the coldest quarter
bio12	Annual precipitation
bio13	Precipitation of the wettest month
bio14	Precipitation of the driest month
bio15	Precipitation seasonality (coefficient of variation)
bio16	Precipitation of the wettest quarter
bio17	Precipitation of the driest quarter
bio18	Precipitation of the warmest quarter
bio19	Precipitation of the coldest quarter

Table S3. Principal components and their Eigenvalues, variance, and cumulative variance for the bioclimatic variables.

PC	Eigenvalue	Variance (%)	Cumulative variance (%)
PC1	10.6	55.9	55.9
PC2	4.8	25.4	81.2
PC3	1.2	6.4	87.6
PC4	0.9	4.9	92.5
PC5	0.7	3.7	96.2
PC6	0.5	2.5	98.7
PC7	0.1	0.7	99.4
PC8	0.1	0.5	99.9
PC9	0.0	0.0	100.0
PC10	0.0	0.0	100.0
PC11	0.0	0.0	100.0
PC12	0.0	0.0	100.0

Table S4. Contributions (loadings) of the bioclimatic variables (Table S2) on each principal component (PC). The darker (greener) the shading, the greater the contribution of a given variable on each PC.

Variable	PC1	PC2	PC3	PC4	PC5
**bio1**	7.0	3.9	0.1	0.1	7.2
**bio2**	0.2	15.3	16.8	0.0	3.1
**bio3**	2.3	11.6	7.9	0.1	3.5
**bio4**	8.4	0.0	0.7	0.1	2.8
**bio5**	0.8	17.1	3.1	1.6	0.0
**bio6**	8.6	0.3	2.5	0.0	5.1
**bio7**	5.9	4.2	7.4	0.9	5.7
**bio8**	1.6	3.3	2.9	59.2	3.7
**bio9**	8.7	0.0	0.2	4.4	0.0
**bio10**	3.0	9.6	0.3	2.7	6.4
**bio11**	8.2	1.6	0.6	0.0	5.1
**bio12**	8.3	1.3	1.5	1.1	3.9
**bio13**	8.4	0.9	0.1	2.2	5.7
**bio14**	1.6	9.8	21.0	0.4	9.3
**bio15**	5.9	0.0	12.8	2.2	20.5
**bio16**	8.4	0.6	0.2	1.9	7.4
**bio17**	3.5	7.0	20.8	1.3	3.4
**bio18**	0.5	13.3	0.7	21.8	1.1
**bio19**	8.6	0.1	0.6	0.0	6.1

Table S5. The best-fit model for CT_{min} and CCRT was defined based on Akaike Information Criterion (AIC) and it was performed in two steps. First, the best-fit fixed effects were defined (highlighted with light grey). Then, the best-fit random effects were defined using the best-fit fixed effects, i.e. this is the final best-fit model used for further analyses (highlighted with dark grey). The model-fit was established using the highest Akaike weight (AICcWt), i.e. probability of being the best model. T refers to temperature during the experiment.

Experiment	Model		df	AICc	ΔAICc	AICcWt
CT_{min}	Model selection for fixed effects	latitude + altitude + latitude:altitude	5	776.51	0.00	0.99
		latitude + altitude	4	786.56	10.05	0.01
		latitude	3	794.84	18.33	0.00
	Model selection for randon effects using the best-fit fixed effects, i.e. the final best-fit model	latitude + altitude + latitude:altitude + experimental batch + T	7	737.54	0.00	0.75
		latitude + altitude + latitude:altitude + experimental batch	6	770.24	32.70	0.00
		latitude + altitude + latitude:altitude + T	6	739.77	2.23	0.25
		latitude + altitude + latitude:altitude (no random effects)	5	776.51	38.97	0.00
CCRT	Model selection for fixed effects	latitude + altitude + latitude:altitude	5	4906.63	0	0.91
		latitude + altitude	4	4913.96	7.32	0.02
		latitude	3	4911.93	5.3	0.06
	Model selection for randon effects using the best-fit fixed effects, i.e. the final best-fit model	latitude + altitude + latitude:altitude + experimental batch + T	7	4910.65	4.02	0.07
		latitude + altitude + latitude:altitude + experimental batch	6	4908.57	1.94	0.2
		latitude + altitude + latitude:altitude + T	6	4908.7	2.07	0.19
		latitude + altitude + latitude:altitude (no random effects)	5	4906.63	0	0.53

Table S6. Pearson correlation test between CT_{min} and CCRT across all populations and separately for each population. Significant correlations are highlighted.

Geographical region	Population	Pearson correlation coefficient	t statistic	df	P value
All regions together	All populations together	0.217	4.04	330	**6.65E-05**
Western coast of NA	Seward	0.387	2.30	30	**0.029**
	Terrace	0.421	2.59	31	**0.015**
	Vancouver	0.190	1.10	32	0.281
	Ashford	0.034	0.19	33	0.848
Rocky Mountains of NA	McBride	-0.112	-0.62	30	0.541
	Crested Butte	0.301	1.73	30	0.094
Fennoscandia	Storslett	0.111	0.62	31	0.540
	Oulanka	-0.102	-0.82	64	0.417
	Korpilahti	0.233	1.37	33	0.179

Table S7. Details of *D. montana* PacBio raw read datasets, which were used to characterize inversions. N50 raw reads = half of the raw reads are larger than or equal to the N50 raw read value.

PacBio reads<br>Region	Population	Strain ID	# reads	# SMRT cells	Bases (Gb)	Estimated Mean coverage	Max read length (bp)	Average read length (bp)	N50 raw read (bp)	Number of reads >= 80 000 bp	Technique	Sequencing facility	Sequencing year	First published
Western coast	Seward	monSE13F37	1,670,083	2	9.9	54.8	168,303	5,906	11,366	132	PacBio Sequel I	Norwegian sequencing center	2018	Poikela et al. (2024)
of North America	Terrace	monTER14F11	328,728	1	3.2	17.9	154,030	9,782	14,605	28	PacBio Sequel II	Novogene	2021	current study
	Ashford	monASH13F9	426,503	1	4.1	23.0	131,309	9,706	13,952	51	PacBio Sequel II	Novogene	2021	current study
	Azalea	monAZA1	233,260	1	2.5	13.8	107,310	10,623	15,435	29	PacBio Sequel II	Novogene	2021	current study
Rocky Mountains	McBride	monMB14F1	347,232	1	3.2	17.6	113,197	9,131	13,176	36	PacBio Sequel II	Novogene	2021	current study
of North America	Jackson	monJX13F48	2,161,617	1	13.9	77.1	111,203	6,422	8,045	19	PacBio Sequel I	BGI	2019	Poikela et al. (2024)
	Crested Butte	mon34CC5	1,281,079	3	7.2	39.9	145,085	5,608	10,260	248	PacBio Sequel II	Novogene	2021	Tahami et al. (2024)
Fennoscandia	Storslett	monSTO20F2	917,273	2	2.8	15.5	146,182	3,046	3,789	177	PacBio Sequel II	Novogene	2021	current study
	Raattama	monRAA20F1	900,722	2	3.0	16.5	133,674	3,304	4,436	190	PacBio Sequel II	Novogene	2021	current study
	Oulanka	monOU13F149	1,431,344	1	8.3	46.4	118,876	5,830	8,836	33	PacBio Sequel I	BGI	2019	Tahami et al. (2024)
Asia	Kamchatka	monKR1309	879,152	1	5.5	30.6	132,961	6,269	9,233	53	PacBio Sequel I	BGI	2019	Tahami et al. (2024)

Table S8. Details of Illumina paired-end raw reads of 101 singly sequenced wild-caught *D. montana* females or their F_1 daughters (continues the next page), used in genomic analyses. The data were first published by Poikela et al. (2024) and Tahami et al. (2024).

Illumina 150bp paired-end reads												
Region	Population	Strain ID	# reads (M)	Bases (Gb)	# lanes	Mean coverage	Library	Technique	Sequencing facility	Sequencing year	Sequenced generation	First published
Western coast of North America	Seward	monSE13F11	80.3	12.0	1	55.3	Truseq	HiSeq X-Ten	Novogene	2021	F_1	Tahami et al. (2024)
		monSE13F12	110.4	16.6	1	60.3	Truseq	HiSeq X-Ten	Novogene	2021	F_1	Tahami et al. (2024)
		monSE13F16	77.3	11.6	1	51.1	Truseq	HiSeq X-Ten	Novogene	2021	F_1	Tahami et al. (2024)
		monSE13F20	80.2	12.0	1	55.5	Truseq	HiSeq X-Ten	Novogene	2021	F_1	Tahami et al. (2024)
		monSE13F21	79.3	11.9	1	55.2	Truseq	HiSeq X-Ten	Novogene	2021	F_1	Tahami et al. (2024)
		monSE13F22	68.2	10.2	1	39.7	Truseq	HiSeq X-Ten	Novogene	2021	F_1	Tahami et al. (2024)
		monSE13F3	71.6	10.7	1	51.1	Truseq	HiSeq X-Ten	Novogene	2021	F_1	Tahami et al. (2024)
		monSE13F37	630.7	95.2	1	434.5	Truseq	HiSeq4000	Norwegian sequencing center	2018	F_1	Poikela et al. (2024)
		monSE13F5	78.6	11.8	1	53.8	Truseq	HiSeq X-Ten	Novogene	2021	F_1	Tahami et al. (2024)
		monSE13F7	79.5	11.9	1	50.9	Truseq	HiSeq X-Ten	Novogene	2021	F_1	Tahami et al. (2024)
		monSE13F9	87.3	13.1	1	49.6	Truseq	HiSeq X-Ten	Novogene	2021	F_1	Tahami et al. (2024)
	Terrace	monTER14F10	76.2	11.4	1	41.6	Truseq	HiSeq X-Ten	Novogene	2021	wild-caught	Tahami et al. (2024)
		monTER14F11	96.8	14.5	1	67.3	Truseq	HiSeq X-Ten	BGI	2019	wild-caught	Poikela et al. (2024)
		monTER14F12	93.4	14.0	1	27.1	Truseq	HiSeq X-Ten	Novogene	2021	wild-caught	Tahami et al. (2024)
		monTER14F13	68.6	10.3	1	44.1	Truseq	HiSeq X-Ten	Novogene	2021	wild-caught	Tahami et al. (2024)
		monTER14F16	88.8	13.3	1	55.5	Truseq	HiSeq X-Ten	Novogene	2021	wild-caught	Tahami et al. (2024)
		monTER14F17	135.6	20.3	2	68.8	Truseq	HiSeq X-Ten	Novogene	2021	wild-caught	Tahami et al. (2024)
		monTER14F19	65.3	9.8	1	34.5	Truseq	HiSeq X-Ten	Novogene	2021	wild-caught	Tahami et al. (2024)
		monTER14F2	76.2	11.4	1	42.3	Truseq	HiSeq X-Ten	Novogene	2021	wild-caught	Tahami et al. (2024)
		monTER14F21	94.0	14.1	1	63.6	Truseq	HiSeq X-Ten	Novogene	2021	wild-caught	Tahami et al. (2024)
		monTER14F24	129.9	19.5	2	72.3	Truseq	HiSeq X-Ten	Novogene	2021	wild-caught	Tahami et al. (2024)
		monTER14F26	65.8	9.9	1	44.5	Truseq	HiSeq X-Ten	Novogene	2021	wild-caught	Tahami et al. (2024)
	Vancouver	monVAN14F1	121.5	18.2	2	70.6	Nextera	HiSeq4000	Edinburgh Genomics	2017	wild-caught	Poikela et al. (2024)
		monVAN14F10	80.5	12.1	1	48.4	Truseq	HiSeq X-Ten	Novogene	2021	wild-caught	Tahami et al. (2024)
		monVAN14F11	65.6	9.8	1	46.4	Truseq	HiSeq X-Ten	Novogene	2021	wild-caught	Tahami et al. (2024)
		monVAN14F17	68.1	10.2	1	43.2	Truseq	HiSeq X-Ten	Novogene	2021	wild-caught	Tahami et al. (2024)
		monVAN14F24	66.6	10.0	1	33.3	Truseq	HiSeq X-Ten	Novogene	2021	wild-caught	Tahami et al. (2024)
		monVAN14F4	86.9	13.0	1	41.7	Truseq	HiSeq X-Ten	Novogene	2021	wild-caught	Tahami et al. (2024)
		monVAN14F5	79.5	11.9	1	44.4	Truseq	HiSeq X-Ten	Novogene	2021	wild-caught	Tahami et al. (2024)
		monVAN14F6	73.8	11.1	1	33.4	Truseq	HiSeq X-Ten	Novogene	2021	wild-caught	Tahami et al. (2024)
		monVAN14F7	84.0	12.6	1	56.3	Truseq	HiSeq X-Ten	Novogene	2021	wild-caught	Tahami et al. (2024)
		monVAN14F8	75.9	11.4	1	40.6	Truseq	HiSeq X-Ten	Novogene	2021	wild-caught	Tahami et al. (2024)
	Ashford	monASH13F11	77.2	11.6	1	38.1	Truseq	HiSeq X-Ten	Novogene	2021	F_1	Tahami et al. (2024)
		monASH13F12	75.8	11.4	1	48.9	Truseq	HiSeq X-Ten	Novogene	2021	F_1	Tahami et al. (2024)
		monASH13F13	167.7	25.2	2	96.3	Nextera	HiSeq4000	Edinburgh Genomics	2017	F_1	Poikela et al. (2024)
		monASH13F17	97.6	14.6	1	71.3	Truseq	HiSeq X-Ten	Novogene	2021	F_1	Tahami et al. (2024)
		monASH13F18	91.9	13.8	1	59.3	Truseq	HiSeq X-Ten	Novogene	2021	F_1	Tahami et al. (2024)
		monASH13F20	92.7	13.9	1	66.7	Truseq	HiSeq X-Ten	Novogene	2021	F_1	Tahami et al. (2024)
		monASH13F4	75.0	11.2	1	52.6	Truseq	HiSeq X-Ten	Novogene	2021	F_1	Tahami et al. (2024)
		monASH13F5	77.7	11.7	1	55.8	Truseq	HiSeq X-Ten	Novogene	2021	F_1	Tahami et al. (2024)
		monASH13F7	76.8	11.5	1	46.4	Truseq	HiSeq X-Ten	Novogene	2021	F_1	Tahami et al. (2024)
		monASH13F9	82.8	12.4	1	50.3	Truseq	HiSeq X-Ten	Novogene	2021	F_1	Tahami et al. (2024)
	Azalea	monAZA1	72.8	10.9	1	52.1	Truseq	HiSeq X-Ten	Novogene	2021	F_1	Tahami et al. (2024)
		monAZA2	94.6	14.2	1	70.7	Truseq	HiSeq X-Ten	BGI	2019	F_1	Poikela et al. (2024)
		monAZA3	90.0	13.5	1	64.1	Truseq	HiSeq X-Ten	Novogene	2021	F_1	Tahami et al. (2024)
Rocky Mountains of North America	McBride	monMB14F1	129.5	19.4	1	53.8	Truseq	HiSeq X-Ten	BGI	2019	wild-caught	Poikela et al. (2024)
		monMB14F15	74.1	11.1	1	52.4	Truseq	HiSeq X-Ten	Novogene	2021	wild-caught	Tahami et al. (2024)
		monMB14F16	65.5	9.8	1	43.5	Truseq	HiSeq X-Ten	Novogene	2021	wild-caught	Tahami et al. (2024)
		monMB14F19	78.9	11.8	1	56.1	Truseq	HiSeq X-Ten	Novogene	2021	wild-caught	Tahami et al. (2024)
		monMB14F22	85.3	12.8	1	61.8	Truseq	HiSeq X-Ten	Novogene	2021	wild-caught	Tahami et al. (2024)
		monMB14F5	67.7	10.2	1	43.5	Truseq	HiSeq X-Ten	Novogene	2021	wild-caught	Tahami et al. (2024)
		monMB14F6	71.4	10.7	1	50.3	Truseq	HiSeq X-Ten	Novogene	2021	wild-caught	Tahami et al. (2024)
		monMB14F7	85.2	12.8	1	60.6	Truseq	HiSeq X-Ten	Novogene	2021	wild-caught	Tahami et al. (2024)
		monMB14F9	74.2	11.1	1	40.0	Truseq	HiSeq X-Ten	Novogene	2021	wild-caught	Tahami et al. (2024)
	Jackson	monJX13F3	130.0	19.5	2	83.4	Nextera	HiSeq4000	Edinburgh Genomics	2017	F_1	Poikela et al. (2024)
		monJX13F41	79.6	11.9	1	51.3	Truseq	HiSeq X-Ten	Novogene	2021	F_1	Tahami et al. (2024)
		monJX13F48	68.0	10.2	1	23.8	Truseq	HiSeq X-Ten	Novogene	2021	F_1	Tahami et al. (2024)
	Crested Butte	mon17CC5	112.2	16.8	2	82.2	Truseq	HiSeq X-Ten	Novogene	2021	F_1	Tahami et al. (2024)
		mon26CC5	79.2	11.9	1	57.1	Truseq	HiSeq X-Ten	Novogene	2021	F_1	Tahami et al. (2024)
		mon29CC5	69.6	10.4	1	49.2	Truseq	HiSeq X-Ten	Novogene	2021	F_1	Tahami et al. (2024)
		mon37CC5	69.5	10.4	1	48.7	Truseq	HiSeq X-Ten	Novogene	2021	F_1	Tahami et al. (2024)
		mon48CC5	94.5	14.2	1	65.3	Truseq	HiSeq X-Ten	Novogene	2021	F_1	Tahami et al. (2024)
		mon4CC5	133.5	20.0	2	95.0	Truseq	HiSeq X-Ten	Novogene	2021	F_1	Tahami et al. (2024)
		mon68CC5	69.9	10.5	1	45.6	Truseq	HiSeq X-Ten	Novogene	2021	F_1	Tahami et al. (2024)
		mon77CC5	76.2	11.4	1	48.7	Truseq	HiSeq X-Ten	Novogene	2021	F_1	Tahami et al. (2024)
		mon81CC5	87.2	13.1	1	18.9	Truseq	HiSeq X-Ten	Novogene	2021	F_1	Tahami et al. (2024)
		mon8CC5	133.3	20.0	2	96.5	Truseq	HiSeq X-Ten	Novogene	2021	F_1	Tahami et al. (2024)

Fennoscandia	Storslett	monSTO20F10	71.9	10.8	1	51.5	Truseq	HiSeq X-Ten	Novogene	2021	F1	Tahami et al. (2024)
		monSTO20F11	109.5	16.4	1	72.5	Truseq	HiSeq X-Ten	Novogene	2021	F1	Tahami et al. (2024)
		monSTO20F13	69.1	10.4	1	50.4	Truseq	HiSeq X-Ten	Novogene	2021	F1	Tahami et al. (2024)
		monSTO20F14	74.9	11.2	1	54.1	Truseq	HiSeq X-Ten	Novogene	2021	F1	Tahami et al. (2024)
		monSTO20F15	66.0	9.9	1	47.6	Truseq	HiSeq X-Ten	Novogene	2021	F1	Tahami et al. (2024)
		monSTO20F2	69.1	10.4	1	11.7	Truseq	HiSeq X-Ten	Novogene	2021	F1	Tahami et al. (2024)
		monSTO20F3	81.3	12.2	1	25.5	Truseq	HiSeq X-Ten	Novogene	2021	F1	Tahami et al. (2024)
		monSTO20F7	92.4	13.9	1	26.5	Truseq	HiSeq X-Ten	Novogene	2021	F1	Tahami et al. (2024)
		monSTO20F8	86.2	12.9	1	33.4	Truseq	HiSeq X-Ten	Novogene	2021	F1	Tahami et al. (2024)
		monSTO20F9	114.6	17.2	1	37.7	Truseq	HiSeq X-Ten	Novogene	2021	F1	Tahami et al. (2024)
	Raattama	monRAA20F1	79.0	11.8	1	51.8	Truseq	HiSeq X-Ten	Novogene	2021	F1	Tahami et al. (2024)
		monRAA20F4	69.7	10.5	1	50.3	Truseq	HiSeq X-Ten	Novogene	2021	F1	Tahami et al. (2024)
		monRAA20F5	65.4	9.8	1	46.4	Truseq	HiSeq X-Ten	Novogene	2021	F1	Tahami et al. (2024)
	Oulanka	monOU13F140	70.7	10.6	1	49.4	Truseq	HiSeq X-Ten	Novogene	2021	F1	Tahami et al. (2024)
		monOU13F149	98.8	14.8	1	72.5	Truseq	HiSeq X-Ten	BGI	2019	F1	Tahami et al. (2024)
		monOU13F163	79.4	11.9	1	33.3	Truseq	HiSeq X-Ten	Novogene	2021	F1	Tahami et al. (2024)
		monOU13F180	77.6	11.6	1	39.3	Truseq	HiSeq X-Ten	Novogene	2021	F1	Tahami et al. (2024)
		monOU13F182	121.9	18.3	1	33.1	Truseq	HiSeq X-Ten	Novogene	2021	F1	Tahami et al. (2024)
		monOU13F199	78.2	11.7	1	34.0	Truseq	HiSeq X-Ten	Novogene	2021	F1	Tahami et al. (2024)
		monOU13F203	68.9	10.3	1	49.5	Truseq	HiSeq X-Ten	Novogene	2021	F1	Tahami et al. (2024)
		monOU13F212	85.3	12.8	1	53.2	Truseq	HiSeq X-Ten	Novogene	2021	F1	Tahami et al. (2024)
		monOU13F216	69.6	10.4	1	44.9	Truseq	HiSeq X-Ten	Novogene	2021	F1	Tahami et al. (2024)
		monOU13F3	70.1	10.5	1	31.6	Truseq	HiSeq X-Ten	Novogene	2021	F1	Tahami et al. (2024)
		monOU13F95	67.9	10.2	1	40.2	Truseq	HiSeq X-Ten	Novogene	2021	F1	Tahami et al. (2024)
	Korpilahti	monKL13F130	110.0	16.5	1	66.5	Truseq	HiSeq X-Ten	Novogene	2021	F1	Tahami et al. (2024)
		monKL13F14	107.2	16.1	1	43.6	Truseq	HiSeq X-Ten	Novogene	2021	F1	Tahami et al. (2024)
		monKL13F20	77.9	11.7	1	25.9	Truseq	HiSeq X-Ten	Novogene	2021	F1	Tahami et al. (2024)
		monKL13F21	68.6	10.3	1	49.8	Truseq	HiSeq X-Ten	Novogene	2021	F1	Tahami et al. (2024)
		monKL13F25	72.7	10.9	2	46.1	Truseq	HiSeq X-Ten	Novogene	2021	F1	Tahami et al. (2024)
		monKL13F50	74.1	11.1	1	50.4	Truseq	HiSeq X-Ten	Novogene	2021	F1	Tahami et al. (2024)
		monKL13F9	93.2	14.0	1	47.6	Truseq	HiSeq X-Ten	Novogene	2021	F1	Tahami et al. (2024)
Asia	Kamchatka	monKR1309	97.9	14.7	1	58.0	Truseq	HiSeq X-Ten	Novogene	2021	~F50	Tahami et al. (2024)
		monKR1314	90.6	13.6	1	45.1	Truseq	HiSeq X-Ten	Novogene	2021	~F50	Tahami et al. (2024)
		monKR1323	70.5	10.6	1	47.7	Truseq	HiSeq X-Ten	Novogene	2021	~F50	Tahami et al. (2024)

Table S9. Details of Illumina pool-sequenced *D. montana* samples (49-50 wild-caught females/males per pool), which were used in characterizing inversion frequences. The data was first published by Wiberg et al. (2021).

Illumina pool-sequence 150bp paired-end reads<br>Region	 Population	Males/females<br>in a pool	 # reads (M)	 Bases (Gb)	Mean<br>coverage	 Technique	 Sequencing facility	Sequencing<br>year	Sequenced<br>generation	 First published
Western coast of	Seward	30/20	406.6	61.0	233.7	Illumina HiSeq3000	Finnish Functional Genomics Centre (Turku, Finland)	2016	wild-caught	Wiberg et al. 2021
North America	Terrace	22/27	231.9	34.8	129.6	Illumina HiSeq3000	Finnish Functional Genomics Centre (Turku, Finland)	2016	wild-caught	Wiberg et al. 2021
	Ashford	16/34	157.1	23.6	99.4	Illumina HiSeq3000	Finnish Functional Genomics Centre (Turku, Finland)	2016	wild-caught	Wiberg et al. 2021
Rocky Mountains of North America	Crested Butte	36/13	204.0	30.6	116.1	Illumina HiSeq3000	Finnish Functional Genomics Centre (Turku, Finland)	2016	wild-caught	Wiberg et al. 2021
Fennoscandia	Oulanka	25/25	168.7	25.3	99.3	Illumina HiSeq3000	Finnish Functional Genomics Centre (Turku, Finland)	2016	wild-caught	Wiberg et al. 2021
	Korpilahti	27/23	179.0	26.9	106.2	Illumina HiSeq3000	Finnish Functional Genomics Centre (Turku, Finland)	2016	wild-caught	Wiberg et al. 2021

Table S10. *D. montana* assembly statistics. The chromosome-level genome was used as a reference genome in the genomic analyses, and the contig-level genomes of five *D. montana* populations were used to characterize inversions. N50 = half of the genome is in contigs larger than or equal to the N50 contig size. N50 count = half of the genome consists of the number indicated in N50 count. The data was first published by Poikela et al. (2024) and Tahami et al. (2024).

Statistic	**chromosome-level genome (Seward)** (Poikela et al. 2024)	**Seward, Alaska monSE13F37** (Poikela et al. 2024)	**Jackson, USA monJX13F48** (Poikela et al. 2024)	**Crested Butte, USA mon34CC5** (Tahami et al. 2024)	**Oulanka, Finland monOU13F149** (Tahami et al. 2024)	**Kamchatka, Russia monKR1309** (Tahami et al. 2024)
**Genome size (Mb)**	145.5	184.3	181.0	175.0	178.9	176.7
**Total contigs**	6	324	796	739	1097	1675
**Longest contig (Mb)**	32.5	29.1	15.2	4.5	3.7	1.2
**N50 (Mb)**	26.5	11.0	1.3	0.8	0.5	0.2
**N50 count**	3	5	20	55	97	253
**GC level (%)**	40.3	40.2	40.2	40.2	40.1	40.1
**Complete BUSCOs (%; n: 3285)**	91.9	98.1	98.5	97.2	96.4	96.0
**Single copy BUSCOs (%)**	91.7	97.6	98.0	96.7	96.0	95.6
**Duplicated BUSCOs (%)**	0.2	0.5	0.5	0.5	0.4	0.4

Table S11. Coordinates and frequencies of the large polymorphic inversion on chromosome 4 (9.5Mb in size). The populations that carry the inversion are highlighted with grey colour.

Coordinates: chromosome 4, 3302717 (proximal breakpoint), 12837900 (distal breakpoint)				Number of individuals with	
Region	Population	Illumina sample	Genotype	the inversion (0/1 or 1/1)	Inversion frequency
Rocky Mountains of North America	McBride	Singly sequenced female: MB14F6	0/1	0.33 (3/9)	0.22 (4/18)
		Singly sequenced female: MB14F7	0/1		
		Singly sequenced female: MB14F19	1/1		
		Other singly sequenced females (6)	0/0		
	Jackson	Singly sequenced female: JX13F3	0/1	0.33 (1/3)	0.17 (1/6)
		Other singly sequenced females (2)	0/0		
	Crested Butte	Singly sequenced female: mon4CC5	1/1	1.00 (59/59)	1.00 (118/118)
		Singly sequenced female: mon8CC5	1/1		
		Singly sequenced female: mon17CC5	1/1		
		Singly sequenced female: mon26CC5	1/1		
		Singly sequenced female: mon29CC5	1/1		
		Singly sequenced female: mon37CC5	1/1		
		Singly sequenced female: mon48CC5	1/1		
		Singly sequenced female: mon68CC5	1/1		
		Singly sequenced female: mon77CC5	1/1		
		Singly sequenced female: mon81CC5	1/1		
		Pool-sequence data (36 males, 13 females)	1/1		
Western coast of North America	Seward	Singly sequenced females (11)	0/0	0.0 (0/61)	0.0 (0/122)
		Pool-sequence data (30 males, 20 females)	0/0		
	Terrace	Singly sequenced females (11)	0/0	0.0 (0/60)	0.0 (0/120)
		Pool-sequence data (22 males, 27 females)	0/0		
	Vancouver	Singly sequenced females (10)	0/0	0.0 (0/10)	0.0 (0/20)
	Ashford	Singly sequenced females (10)	0/0	0.0 (0/60)	0.0 (0/120)
		Pool-sequence data (16 males, 34 females)	0/0		
	Azalea	Singly sequenced females (3)	0/0	0.0 (0/3)	0.0 (0/6)
Fennoscandia	Storslett	Singly sequenced females (10)	0/0	0.0 (0/10)	0.0 (0/20)
	Raattama	Singly sequenced females (3)	0/0	0.0 (0/3)	0.0 (0/6)
	Oulanka	Singly sequenced females (11)	0/0	0.0 (0/61)	0.0 (0/122)
		Pool-sequence data (25 males, 25 females)	0/0		
	Korpilahti	Singly sequenced females (7)	0/0	0.0 (0/57)	0.0 (0/114)
		Pool-sequence data (27 males, 23 females)	0/0		
Asia	Kamchatka	Singly sequenced females (3)	0/0	0.0 (0/3)	0.0 (0/6)

Table S12. Coordinates and frequencies of the small polymorphic inversion on chromosome 4 (~1500bp in size). The populations that carry the inversion are highlighted with grey colour.

Coordinates: chromosome 4, 1748864 (proximal breakpoint), 1750399 (distal breakpoint)				Number of individuals with	
Region	Population	Illumina sample	Genotype	the inversion (0/1 or 1/1)	Inversion frequency
Rocky Mountains of North America	McBride	Singly sequenced females (9)	0/0	0.0 (0/9)	0.0 (0/18)
	Jackson	Singly sequenced females (3)	0/0	0.0 (0/3)	0.0 (0/6)
	Crested Butte	Singly sequenced females (10)	0/0	0.0 (0/59)	0.0 (0/118)
		Pool-sequence data (36 males, 13 females)	0/0		
Western coast of North America	Seward	Singly sequenced females (11)	0/0	0.0 (0/61)	0.0 (0/122)
		Pool-sequence data (30 males, 20 females)	0/0		
	Terrace	Singly sequenced females (11)	0/0	0.0 (0/60)	0.0 (0/120)
		Pool-sequence data (22 males, 27 females)	0/0		
	Vancouver	Singly sequenced females (10)	0/0	0.0 (0/10)	0.0 (0/20)
	Ashford	Singly sequenced females (10)	0/0	0.0 (0/60)	0.0 (0/120)
		Pool-sequence data (16 males, 34 females)	0/0		
	Azalea	Singly sequenced females (3)	0/0	0.0 (0/3)	0.0 (0/6)
Fennoscandia	Storslett	Singly sequenced females (10)	1/1	1.0 (10/10)	1.0 (20/20)
	Raattama	Singly sequenced females (3)	1/1	1.0 (3/3)	1.0 (6/6)
	Oulanka	Singly sequenced females (11)	1/1	1.0 (61/61)	1.0 (122/122)
		Pool-sequence data (25 males, 25 females)	1/1		
	Korpilahti	Singly sequenced females (7)	1/1	1.0 (57/57)	1.0 (114/114)
		Pool-sequence data (27 males, 23 females)	1/1		
Asia	Kamchatka	Singly sequenced females (3)	1/1	1.0 (3/3)	1.0 (6/6)

Table S13. Eigenvalues, variance (%) and cumulative variance (%) of each principal component (PC) were calculated for filtered and pruned SNPs obtained from 101 Illumina re-sequence *D. montana* samples. The analyses were performed on different data partitions: all chromosomes, the polymorphic chromosome 4 inversion, non-inverted chromosome 4, and each chromosome separately.

Genomic region	# SNPs		PC1	PC2	PC3	PC4	PC5	PC6	PC7	PC8	PC9	PC10	PC11	PC12	PC13	PC14	PC15	PC16	PC17	PC18	PC19	PC20
**All chromosomes**	1,986,534	**Eigenvalue**	19.9	12.3	3.3	2.6	1.8	1.6	1.5	1.4	1.2	1.2	1.1	1.1	1.1	1.0	1.0	1.0	1.0	1.0	1.0	1.0
		**Variance (%)**	34.9	21.6	5.7	4.6	3.2	2.8	2.7	2.4	2.1	2.0	2.0	1.9	1.8	1.8	1.8	1.8	1.8	1.8	1.7	1.7
		**Cumulative variance (%)**	34.9	56.5	62.2	66.8	69.9	72.8	75.4	77.8	79.9	81.9	83.9	85.7	87.6	89.4	91.2	93.0	94.8	96.5	98.3	100.0
**Chromosome 4 inversion**	166,193	**Eigenvalue**	19.8	13.3	6.4	5.3	2.9	2.6	2.3	1.3	1.2	1.2	1.1	1.1	1.0	1.0	1.0	1.0	1.0	1.0	1.0	1.0
		**Variance (%)**	29.8	20.0	9.6	8.0	4.3	4.0	3.4	2.0	1.8	1.8	1.7	1.7	1.6	1.5	1.5	1.5	1.5	1.5	1.5	1.5
		**Cumulative variance (%)**	29.8	49.8	59.4	67.3	71.6	75.6	79.0	81.0	82.8	84.6	86.3	88.0	89.5	91.1	92.6	94.1	95.6	97.1	98.5	100.0
**Chromosome 4 colinear**	314,473	**Eigenvalue**	21.5	11.7	7.6	3.0	2.5	2.4	2.0	1.8	1.6	1.5	1.3	1.2	1.2	1.1	1.1	1.1	1.0	1.0	1.0	1.0
		**Variance (%)**	32.3	17.5	11.4	4.5	3.8	3.6	3.0	2.8	2.3	2.2	2.0	1.9	1.7	1.7	1.7	1.6	1.5	1.5	1.5	1.5
		**Cumulative variance (%)**	32.3	49.8	61.2	65.8	69.6	73.2	76.2	78.9	81.3	83.5	85.4	87.3	89.0	90.7	92.4	94.0	95.6	97.1	98.5	100.0
**Chromosome 2L**	266,896	**Eigenvalue**	20.0	12.4	3.0	1.9	1.7	1.5	1.4	1.3	1.1	1.1	1.1	1.1	1.0	1.0	1.0	1.0	1.0	1.0	1.0	1.0
		**Variance (%)**	35.8	22.3	5.4	3.3	3.1	2.8	2.5	2.3	2.0	1.9	1.9	1.9	1.9	1.9	1.9	1.8	1.8	1.8	1.8	1.8
		**Cumulative variance (%)**	35.8	58.1	63.5	66.9	70.0	72.8	75.3	77.6	79.5	81.5	83.4	85.3	87.1	89.0	90.9	92.7	94.5	96.4	98.2	100.0
**Chromosome 2R**	131,003	**Eigenvalue**	19.7	12.3	2.8	1.9	1.3	1.2	1.1	1.1	1.1	1.0	1.0	1.0	1.0	1.0	1.0	1.0	1.0	1.0	1.0	1.0
		**Variance (%)**	36.7	23.0	5.3	3.5	2.4	2.3	2.0	2.0	2.0	2.0	1.9	1.9	1.9	1.9	1.9	1.9	1.9	1.9	1.9	1.9
		**Cumulative variance (%)**	36.7	59.6	64.9	68.4	70.9	73.1	75.1	77.1	79.1	81.1	83.0	84.9	86.8	88.7	90.6	92.5	94.4	96.3	98.1	100.0
**Chromosome 3**	398,598	**Eigenvalue**	19.1	12.2	2.6	1.6	1.3	1.2	1.2	1.1	1.1	1.0	1.0	1.0	1.0	1.0	1.0	1.0	1.0	1.0	1.0	1.0
		**Variance (%)**	36.2	23.1	5.0	3.0	2.5	2.4	2.2	2.1	2.0	2.0	2.0	2.0	2.0	2.0	2.0	2.0	2.0	1.9	1.9	1.9
		**Cumulative variance (%)**	36.2	59.3	64.2	67.2	69.7	72.1	74.3	76.4	78.4	80.4	82.4	84.4	86.3	88.3	90.3	92.2	94.2	96.1	98.1	100.0
**Chromosome 4**	502,781	**Eigenvalue**	20.7	12.1	6.3	3.7	3.2	2.5	2.0	1.6	1.5	1.3	1.3	1.2	1.2	1.2	1.1	1.0	1.0	1.0	1.0	1.0
		**Variance (%)**	31.4	18.4	9.6	5.7	4.8	3.8	3.0	2.5	2.3	2.0	2.0	1.8	1.8	1.7	1.7	1.6	1.5	1.5	1.5	1.4
		**Cumulative variance (%)**	31.4	49.8	59.4	65.1	69.9	73.7	76.7	79.2	81.4	83.5	85.5	87.3	89.1	90.9	92.5	94.1	95.6	97.1	98.6	100.0
**Chromosome 5**	403,222	**Eigenvalue**	19.7	12.5	2.8	1.6	1.3	1.2	1.2	1.2	1.1	1.0	1.0	1.0	1.0	1.0	1.0	1.0	1.0	1.0	1.0	1.0
		**Variance (%)**	36.6	23.3	5.2	2.9	2.5	2.3	2.2	2.1	2.0	1.9	1.9	1.9	1.9	1.9	1.9	1.9	1.9	1.9	1.9	1.9
		**Cumulative variance (%)**	36.6	59.8	65.1	68.0	70.5	72.8	75.0	77.1	79.1	81.0	82.9	84.9	86.8	88.7	90.6	92.5	94.4	96.2	98.1	100.0
**Chromosome X**	284,034	**Eigenvalue**	21.8	12.6	6.6	2.8	1.9	1.8	1.3	1.2	1.1	1.1	1.1	1.1	1.1	1.1	1.1	1.1	1.1	1.1	1.1	1.1
		**Variance (%)**	34.5	19.9	10.4	4.4	3.0	2.8	2.1	2.0	1.8	1.8	1.8	1.7	1.7	1.7	1.7	1.7	1.7	1.7	1.7	1.7
		**Cumulative variance (%)**	34.5	54.4	64.8	69.3	72.2	75.0	77.1	79.1	80.9	82.7	84.4	86.2	87.9	89.6	91.4	93.1	94.8	96.6	98.3	100.0

Table S14. Mean genetic diversity (π) of *D. montana* populations. The darker (greener) the colour, the greater the value.

		Intergenic		Intronic		Coding	
**Continent**	**Region**	**Population**	**π**	**Population**	**π**	**Population**	**π**
**North America**	**Western coast**	Seward	0.00923	Seward	0.00994	Seward	0.00533
		Terrace	0.00977	Terrace	0.01050	Terrace	0.00558
		Vancouver	0.00951	Vancouver	0.01022	Vancouver	0.00543
		Ashford	0.00947	Ashford	0.01015	Ashford	0.00541
		Azalea	0.00951	Azalea	0.01015	Azalea	0.00542
	**Rocky Mountains**	McBride**	0.00967	McBride**	0.01036	McBride**	0.00546
		Jackson**	0.01005	Jackson**	0.01077	Jackson**	0.00577
		Crested Butte*	0.00436	Crested Butte*	0.00463	Crested Butte*	0.00256
**Asia**	**Kamchatka**	Kamchatka†	0.00447	Kamchatka†	0.00477	Kamchatka†	0.00255
**Europe**	**North Europe**	Storslett	0.00679	Storslett	0.00723	Storslett	0.00400
		Raattama	0.00675	Raattama	0.00719	Raattama	0.00397
		Oulanka	0.00678	Oulanka	0.00723	Oulanka	0.00399
		Korpilahti	0.00652	Korpilahti	0.00700	Korpilahti	0.00388

* Inversion fixed

** Inversion at lower frequency

† Samples were collected from isofemale strains kept in the laboratory for ~50 generations.

Table S15. Mean genetic diversity (π) of *D. montana* populations and mean genetic divergence (d_{xy}) and genetic differentiation (f_{st}) between Crested Butte (inversion fixed*) and the other populations (inversion absent, or at lower frequency**) in inverted and colinear regions. The darker the colour, the greater the value.

Genetic diversity		π INV	π COL
	Seward	0.01151	0.00861
	Terrace	0.01173	0.00901
	Vancouver	0.01153	0.00882
	Ashford	0.01127	0.00861
	Azalea	0.01103	0.00844
	McBride**	0.01243	0.00895
	Jackson**	0.01229	0.00924
	Crested Butte*	0.00428	0.00414
	Kamchatka	0.00666	0.00502
	Storslett	0.00862	0.0062
	Raattama	0.00852	0.00619
	Oulanka	0.00871	0.00621
	Korpilahti	0.00833	0.00596
**Genetic divergence**		**dxy INV**	**dxy COL**
Crested Butte* vs	Seward	0.01596	0.01102
	Terrace	0.01594	0.01097
	Vancouver	0.01581	0.01097
	Ashford	0.01583	0.01097
	Azalea	0.01593	0.01112
	McBride**	0.01502	0.01091
	Jackson**	0.01505	0.01091
	Kamchatka	0.01601	0.01137
	Storslett	0.01599	0.01143
	Raattama	0.01607	0.01147
	Oulanka	0.01605	0.01145
	Korpilahti	0.01603	0.01146
**Genetic differentiation**		**fst INV**	**fst COL**
Crested Butte* vs	Seward	0.371	0.277
	Terrace	0.349	0.257
	Vancouver	0.350	0.266
	Ashford	0.356	0.267
	Azalea	0.343	0.274
	McBride**	0.317	0.259
	Jackson**	0.302	0.244
	Kamchatka	0.527	0.471
	Storslett	0.430	0.380
	Raattama	0.436	0.384
	Oulanka	0.428	0.383
	Korpilahti	0.442	0.392

Table S16. BayeScEnv was used to identify loci associated with environmental variables (PC1 and PC2) and cold phenotypes (CT_{min}, CCRT). The number and proportion of significant SNPs associated with a given variable were obtained from significance level 0.05 and are shown separately for each chromosome and chromosome arm, and for the large inversion on chromosome 4 (4INV = inversion using the strict inversion breakpoints, 4INV+1.5Mb = inversion extends 1.5Mb beyond the breakpoints into the colinear regions), and for colinear regions (COL = colinear regions using the strict inversion breakpoints, COL-1.5Mb = colinear regions except 1.5Mb regions beyond the breakpoints that were included as part of the inversion).

Variable	Genomic region	# Significant SNPs	# Non-significant SNPs	# SNPs total	% Significant SNPs
PC1	All	66852	3049251	3546748	1.88
	2L	6131	430645	436776	1.40
	2R	2955	224029	226984	1.30
	3	5186	682366	687552	0.75
	4	15658	965111	980769	1.60
	5	9033	648757	657790	1.37
	X	27889	528988	556877	5.01
	4INV	6181	311867	318048	1.94
	COL	60671	3168029	3228700	1.88
	4INV+1.5Mb	9095	378563	387658	2.35
	COL-1.5Mb	57757	3101333	3159090	1.83
PC2	All	34481	3075920	3545139	0.97
	2L	1766	434738	436504	0.40
	2R	804	226105	226909	0.35
	3	1709	685594	687303	0.25
	4	9188	971395	980583	0.94
	5	2205	655236	657441	0.34
	X	18809	537590	556399	3.38
	4INV	5501	312525	318026	1.73
	COL	28980	3198133	3227113	0.90
	4INV+1.5Mb	7746	379849	387595	2.00
	COL-1.5Mb	26735	3130809	3157544	0.85
CTmin	All	54789	3051662	3538127	1.55
	2L	4015	431676	435691	0.92
	2R	1802	224731	226533	0.80
	3	3345	683367	686712	0.49
	4	15267	963093	978360	1.56
	5	5004	651129	656133	0.76
	X	25356	529342	554698	4.57
	4INV	2802	314419	317221	0.88
	COL	51987	3168919	3220906	1.61
	4INV+1.5Mb	4380	382076	386456	1.13
	COL-1.5Mb	50409	3101262	3151671	1.60
CCRT	All	32464	3078639	3546464	0.92
	2L	1396	435361	436757	0.32
	2R	776	226190	226966	0.34
	3	1235	686300	687535	0.18
	4	6374	974456	980830	0.65
	5	1894	655863	657757	0.29
	X	20789	535830	556619	3.73
	4INV	905	317301	318206	0.28
	COL	31559	3196699	3228258	0.98
	4INV+1.5Mb	1494	386358	387852	0.39
	COL-1.5Mb	30970	3127642	3158612	0.98

Table S17. Chi Square (X^2) test was used to analyse BayeScEnv results between the large inversion on chromosome 4 (4INV = inversion using the strict inversion breakpoints, 4INV+1.5Mb = inversion extends 1.5Mb beyond the breakpoints into the colinear regions) and colinear regions (COL = colinear regions using the strict inversion breakpoints, COL-1.5Mb = colinear regions except 1.5Mb regions beyond the breakpoints that were included as part of the inversion). Significant P-values are bolded, and significant comparisons in the expected direction (4INV>COL) are highlighted with grey shading.

Chi Square test						
	Genomic region	# Significant SNPs	# Non-significant SNPs	% significant SNPs	X^2	P-value
**PC1**	**COL**	60671	3168029	1.88	6.47	**0.011**
	**4INV**	6181	311867	1.94		
	**COL-1.5Mb**	57757	3101333	1.83	500.71	**<0.001**
	**4INV+1.5Mb**	9095	378563	2.35		
**PC2**	**COL**	28980	3198133	0.90	2079.18	**<0.001**
	**4INV**	5501	312525	1.73		
	**COL-1.5Mb**	26735	3130809	0.85	4754.75	**<0.001**
	**4INV+1.5Mb**	7746	379849	2.00		
**CTmin**	**COL**	51987	3168919	1.61	1011.50	**<0.001**
	**4INV**	2802	314419	0.88		
	**COL-1.5Mb**	50409	3101262	1.60	490.47	**<0.001**
	**4INV+1.5Mb**	4380	382076	1.13		
**CCRT**	**COL**	31559	3196699	0.98	1534.47	**<0.001**
	**4INV**	905	317301	0.28		
	**COL-1.5Mb**	30970	3127642	0.98	1349.64	**<0.001**
	**4INV+1.5Mb**	1494	386358	0.39		

Table S18. The table shows the number of genes that were associated with the significant SNPs linked to climate variability (PC1, PC2) and cold tolerance (CT_{min}, CCRT) within the inversion (Table S17, full lists of genes in Supplementary file 1). It also lists the GO-terms in which the genes showed a significant enrichment. We used orthologs from both *D. virilis* and *D. melanogaster*, as *D. virilis* and *D. montana* are more closely related, but *D. melanogaster* has superior annotation. 4INV indicates the genes located within the inversion using the strict inversion breakpoints, and 4INV+1.5Mb the genes located within the inversion or within 1.5Mb regions beyond the breakpoints into the colinear regions.

Variable	Region	# genes	GO-term	Species	Benjamini corrected P-value
PC1	4INV	392	EGF-like_domain	*D. virilis*	1.12E-02
			EGF	*D. virilis*	2.66E-02
			EGF-like_domain	*D. melanogaster*	2.82E-05
			membrane	*D. melanogaster*	1.78E-04
			EGF	*D. melanogaster*	4.00E-04
			phagocytosis	*D. melanogaster*	2.19E-03
			EGF-like_dom	*D. melanogaster*	3.95E-03
			voltage-gated calcium channel complex	*D. melanogaster*	2.98E-02
			DOMAIN:EGF-like	*D. melanogaster*	3.45E-02
			sodium ion transport	*D. melanogaster*	4.02E-02
			Transmembrane	*D. melanogaster*	4.05E-02
			DOMAIN:Ig-like	*D. melanogaster*	4.43E-02
			Signal-anchor	*D. melanogaster*	4.51E-02
PC1	4INV+1.5Mb	507	EGF-like_domain	*D. melanogaster*	1.68E-04
			phagocytosis	*D. melanogaster*	1.12E-03
			EGF	*D. melanogaster*	5.25E-03
			EGF-like_dom	*D. melanogaster*	4.61E-02
PC2	4INV	332	EGF-like_domain	*D. virilis*	1.10E-04
			EGF	*D. virilis*	1.15E-03
			EGF-like_dom	*D. virilis*	6.81E-03
			IG	*D. virilis*	2.44E-02
			Ig-like_dom	*D. virilis*	3.84E-02
			Ig-like_dom_sf	*D. virilis*	3.84E-02
			Neural/epithelial_adhesion	*D. virilis*	3.84E-02
			Ig_sub	*D. virilis*	3.84E-02
			DOMAIN:EGF-like	*D. virilis*	4.75E-02
			DOMAIN:Ig-like	*D. virilis*	4.75E-02
			EGF-like_domain	*D. melanogaster*	8.22E-08
			EGF	*D. melanogaster*	9.44E-06
			phagocytosis	*D. melanogaster*	2.21E-05
			EGF-like_dom	*D. melanogaster*	6.37E-05
			membrane	*D. melanogaster*	4.91E-04
			DOMAIN:EGF-like	*D. melanogaster*	9.84E-04
			Growth_fac_rcpt_cys_sf	*D. melanogaster*	1.28E-02
			DOMAIN:Ig-like	*D. melanogaster*	4.13E-02
			Ig-like_dom_sf	*D. melanogaster*	4.26E-02
			Ig-like_dom	*D. melanogaster*	4.26E-02
			Neural/epithelial_adhesion	*D. melanogaster*	4.26E-02
PC2	4INV+1.5Mb	423	EGF-like_domain	*D. virilis*	6.32E-04
			EGF	*D. virilis*	1.16E-02
			EGF-like_domain	*D. melanogaster*	6.15E-07
			membrane	*D. melanogaster*	9.56E-05
			EGF	*D. melanogaster*	1.66E-04
			phagocytosis	*D. melanogaster*	1.87E-04
			EGF-like_dom	*D. melanogaster*	9.83E-04
			Growth_fac_rcpt_cys_sf	*D. melanogaster*	6.70E-03
			DOMAIN:EGF-like	*D. melanogaster*	1.03E-02

Variable	Region	# genes	GO-term	Species	Benjamini corrected P-value
CT_{min}	4INV	307	EGF	*D. virilis*	2.67E-02
			IG	*D. virilis*	4.88E-02
			membrane	*D. melanogaster*	5.61E-05
			Transmembrane	*D. melanogaster*	1.99E-03
			Ion channel	*D. melanogaster*	2.62E-03
			EGF	*D. melanogaster*	3.49E-03
			voltage-gated calcium channel complex	*D. melanogaster*	1.13E-02
			TRANSMEM:Helical	*D. melanogaster*	2.70E-02
			DOMAIN:EGF-like	*D. melanogaster*	2.70E-02
CT_{min}	4INV+1.5Mb	400	membrane	*D. melanogaster*	1.95E-05
			Transmembrane	*D. melanogaster*	3.64E-03
			voltage-gated calcium channel complex	*D. melanogaster*	3.15E-02
			EGF	*D. melanogaster*	3.16E-02
			TRANSMEM:Helical	*D. melanogaster*	3.32E-02
CCRT	4INV	146	voltage-gated calcium channel complex	*D. virilis*	2.84E-03
			Calcium transport	*D. virilis*	3.92E-03
			FA58C	*D. virilis*	3.00E-02
			IG	*D. virilis*	3.00E-02
			DOMAIN:F5/8 type C	*D. virilis*	3.39E-02
			DOMAIN:Ig-like	*D. virilis*	3.39E-02
			TRANSMEM:Helical	*D. virilis*	3.39E-02
			Transmembrane	*D. virilis*	3.86E-02
			Membrane	*D. virilis*	4.71E-02
			membrane	*D. melanogaster*	2.47E-04
			Transmembrane	*D. melanogaster*	4.19E-04
			voltage-gated calcium channel complex	*D. melanogaster*	7.58E-04
			TRANSMEM:Helical	*D. melanogaster*	1.03E-03
			voltage-gated calcium channel activity	*D. melanogaster*	4.83E-03
			Receptor	*D. melanogaster*	9.74E-03
			Transmembrane helix	*D. melanogaster*	1.41E-02
			Membrane	*D. melanogaster*	1.71E-02
			Calcium transport	*D. melanogaster*	1.72E-02
			EGF	*D. melanogaster*	3.40E-02
			FA58C	*D. melanogaster*	3.51E-02
CCRT	4INV+1.5Mb	186	Calcium transport	*D. virilis*	1.20E-02
			voltage-gated calcium channel complex	*D. virilis*	4.80E-03
			Transmembrane	*D. virilis*	9.63E-04
			TRANSMEM:Helical	*D. virilis*	6.57E-03
			DOMAIN:Sodium/calcium exchanger mem	*D. virilis*	1.62E-02
			Antiport	*D. virilis*	2.36E-02
			DOMAIN:F5/8 type C	*D. virilis*	3.8E-02
			Transmembrane	*D. melanogaster*	7.62E-05
			membrane	*D. melanogaster*	4.46E-04
			TRANSMEM:Helical	*D. melanogaster*	6.83E-04
			voltage-gated calcium channel complex	*D. melanogaster*	2.01E-03
			Calcium transport	*D. melanogaster*	4.54E-03
			Receptor	*D. melanogaster*	7.62E-03
			Transmembrane helix	*D. melanogaster*	7.67E-03
			voltage-gated calcium channel activity	*D. melanogaster*	1.19E-02

Table S19. Support, measured as ΔlnCL, and parameter estimates for divergence time (*T* in years/generations), migration rate (*m*) and effective population sizes (N_e) for three *D. montana* populations and their common ancestral population under strict divergence model (DIV, *m*=0) and isolation with migration models with both gene flow directions ($IM_{A\text{->}B}$ or $IM_{B\text{->}A}$). Migration rate (*m*) estimates correspond to M (=$4N_e m$) individuals per generation (forwards in time). The model comparisons are based on 256bp blocks and were performed for colinear non-repetitive intergenic regions to minimize the direct effects of selection. Grey shading indicates the best-fit model for each comparison (see Fig. S12).

Population	Model	Ancestral *Ne*	Pop. *A Ne*	Pop. *B Ne*	T (years)	*m (M = 4Ne m)*	ΔlnCL
Crested Butte (A) - Vancouver (B)	DIV	620,765	205,734	1,181,914	594,424	-	1,093,392
	IM *D. monA --> D. monB*	617,355	207,830	1,158,007	610,170	2.60E-08 (0.12)	1,089,932
	IM *D. monB --> D. monA*	438,089	147,816	1,070,603	958,812	4.95E-07 (0.29)	0
Crested Butte (A) - Storslett (B)	DIV	601,013	228,554	437,696	729,495	-	646,726
	IM *D. monA --> D. monB*	586,550	232,831	431,550	772,769	3.92E-08 (0.07)	615,447
	IM *D. monB --> D. monA*	474,766	198,026	471,474	1,016,841	2.19E-07 (0.17)	0
Storslett (A) - Vancouver (B)	DIV	527,819	413,484	1,228,026	651,134	-	403,580
	IM *D. monA --> D. monB*	460,630	432,335	917,162	899,273	3.88E-07 (1.42)	17,628
	IM *D. monB --> D. monA*	441,187	311,827	1,096,621	861,097	4.83E-07 (0.60)	0

Table S20. Barrier regions (Mb) and barrier fraction (%) for different chromosomes, for the large inversion on chromosome 4 (4INV = inversion using the strict inversion breakpoints, 4INV+1.5Mb = inversion extends 1.5Mb beyond the breakpoints into the colinear regions), and for colinear regions (COL = colinear regions using the strict inversion breakpoints, COL-1.5Mb = colinear regions except 1.5Mb regions beyond the breakpoints that were included as part of the inversion).

Genomic region	Barrier (Mb)	Total (Mb)	Barrier fraction (%)
**All**	15.57	145.45	10.71
**X**	0.32	29.14	1.09
**2L**	1.28	20.25	6.32
**2R**	0.78	11.00	7.12
**3**	1.33	26.02	5.11
**4**	8.20	32.54	25.19
**5**	3.67	26.51	13.84
**4INV**	6.00	9.54	62.91
**4INV + 1.5Mb**	6.83	12.54	54.52
**COL**	9.58	135.92	7.05
**COL - 1.5MB**	8.74	132.92	6.58

Table S21. A Chi-Square (X^2) test was used to estimate barrier enrichment in inverted regions (4INV = inversion using the strict inversion breakpoints, 4INV+1.5Mb = inversion extends 1.5Mb beyond the breakpoints into the colinear regions) compared to colinear regions (COL = colinear regions using the strict inversion breakpoints, COL-1.5Mb = colinear regions except 1.5Mb regions beyond the breakpoints that were included as part of the inversion). The test was based on the number of non-overlapping barrier and non-barrier windows (divided by five to obtain non-overlapping windows) in each region.

Chi-square test		#barrier windows	#non-barrier windows	X^2-statistic	P-value
**Genomic region**	**4INV**	45.6	62	309.9	**<0.0001**
	**COL**	47.6	1621.6		
	**4INV+1.5MB**	51.2	73.8	337.4	**<0.0001**
	**COL-1.5MB**	42	1609.8		

Supplementary Figures

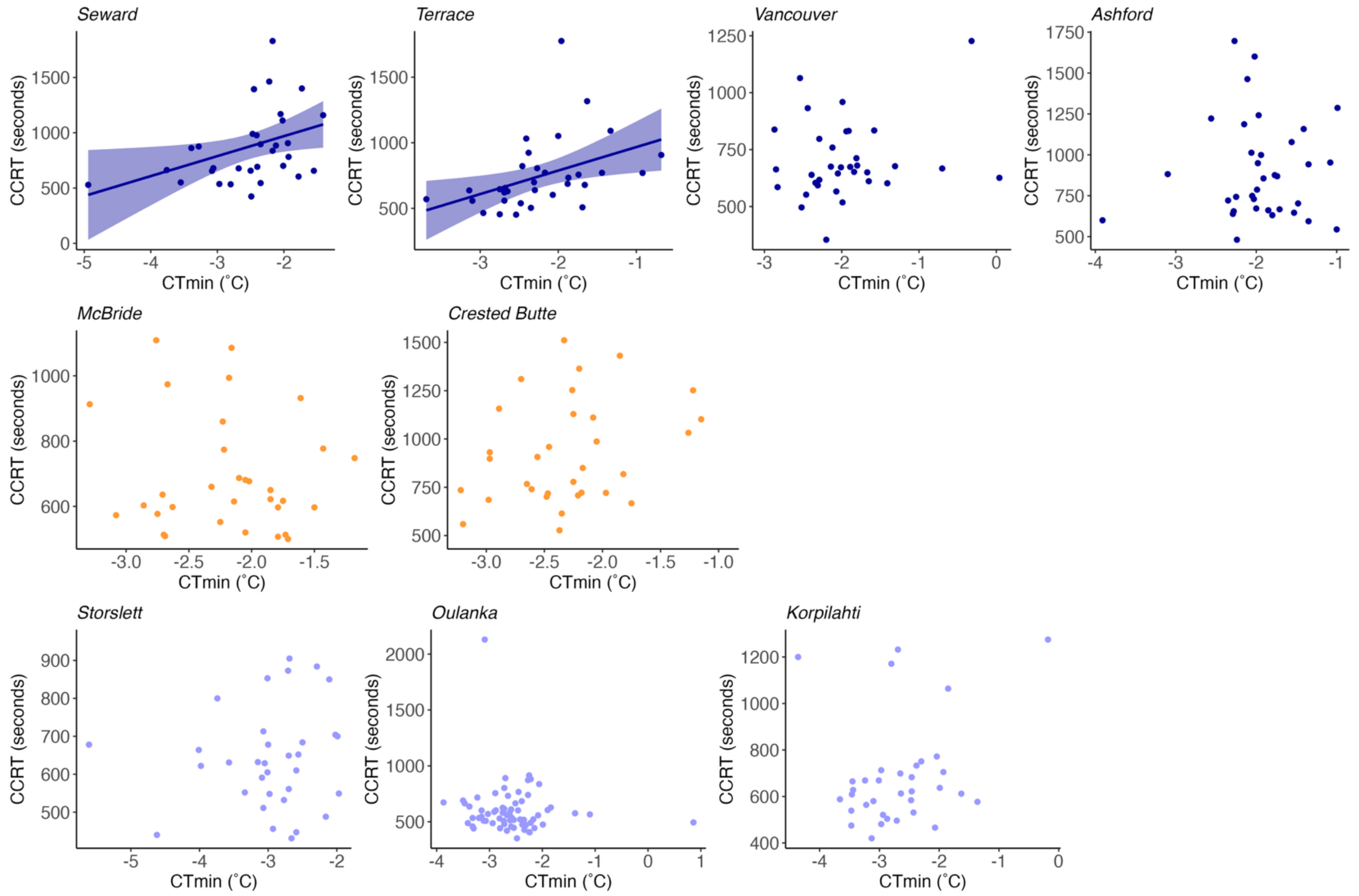

Figure S1. Pearson correlation between CT_{min} and CCRT in nine *D. montana* populations (black dashed line). Regression lines indicate significant correlations. The respective statistics are shown in Table S6.

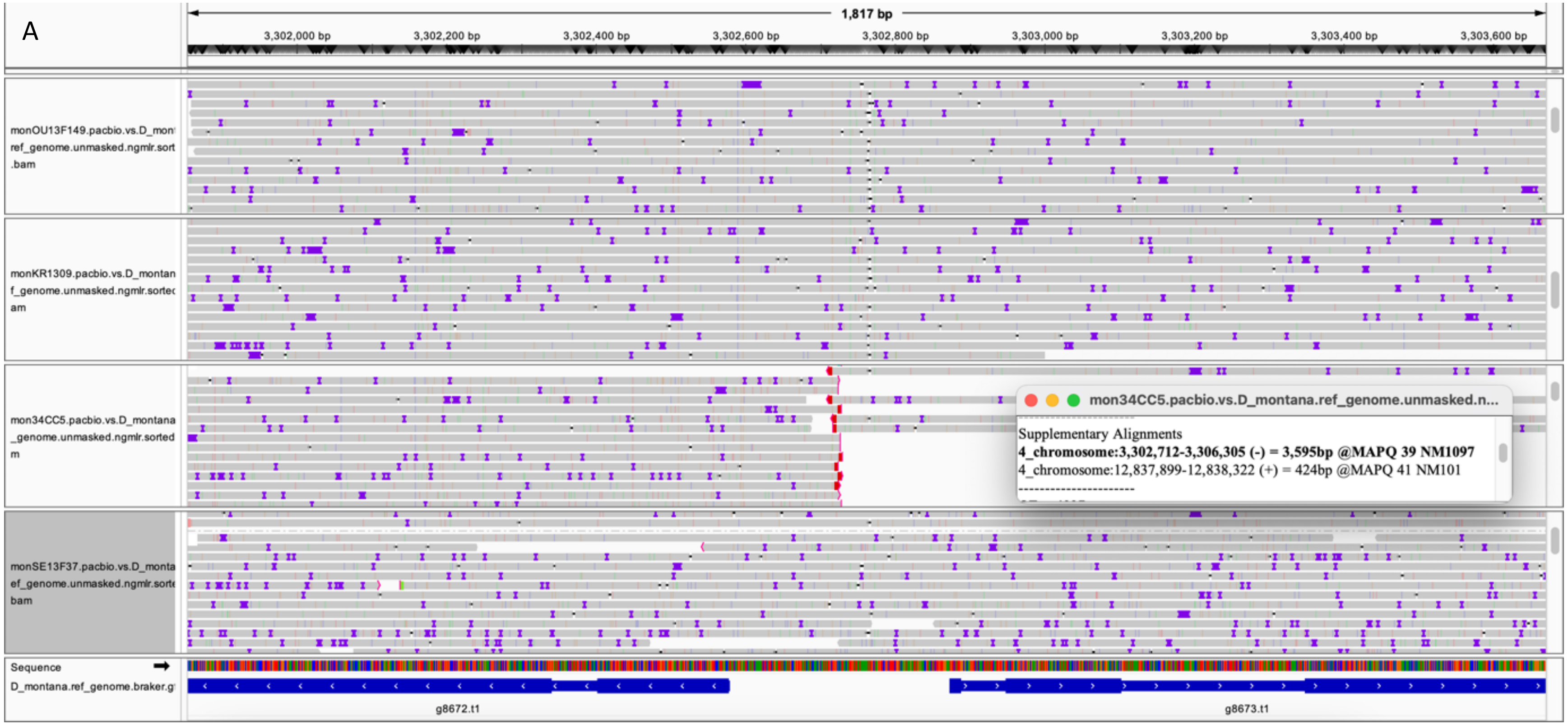

A
1,817 bp
3,302,000 bp
3,302,200 bp
3,302,400 bp
3,302,600 bp
3,302,800 bp
3,303,000 bp
3,303,200 bp
3,303,400 bp
3,303,600 bp
monOU13F149.pacbio.vs.D_mon ref_genome.unmasked.ngmlr.sort .bam
monKR1309.pacbio.vs.D_montan f_genome.unmasked.ngmlr.sorted am
mon34CC5.pacbio.vs.D_montana _genome.unmasked.ngmlr.sorted m
monSE13F37.pacbio.vs.D_monta ef_genome.unmasked.ngmlr.sorte bam
mon34CC5.pacbio.vs.D_montana.ref_genome.unmasked.n...
Supplementary Alignments
4_chromosome:3,302,712-3,306,305 (-) = 3,595bp @MAPQ 39 NM1097
4_chromosome:12,837,899-12,838,322 (+) = 424bp @MAPQ 41 NM101
Sequence
D_montana.ref_genome.braker.g
g8672.t1
g8673.t1

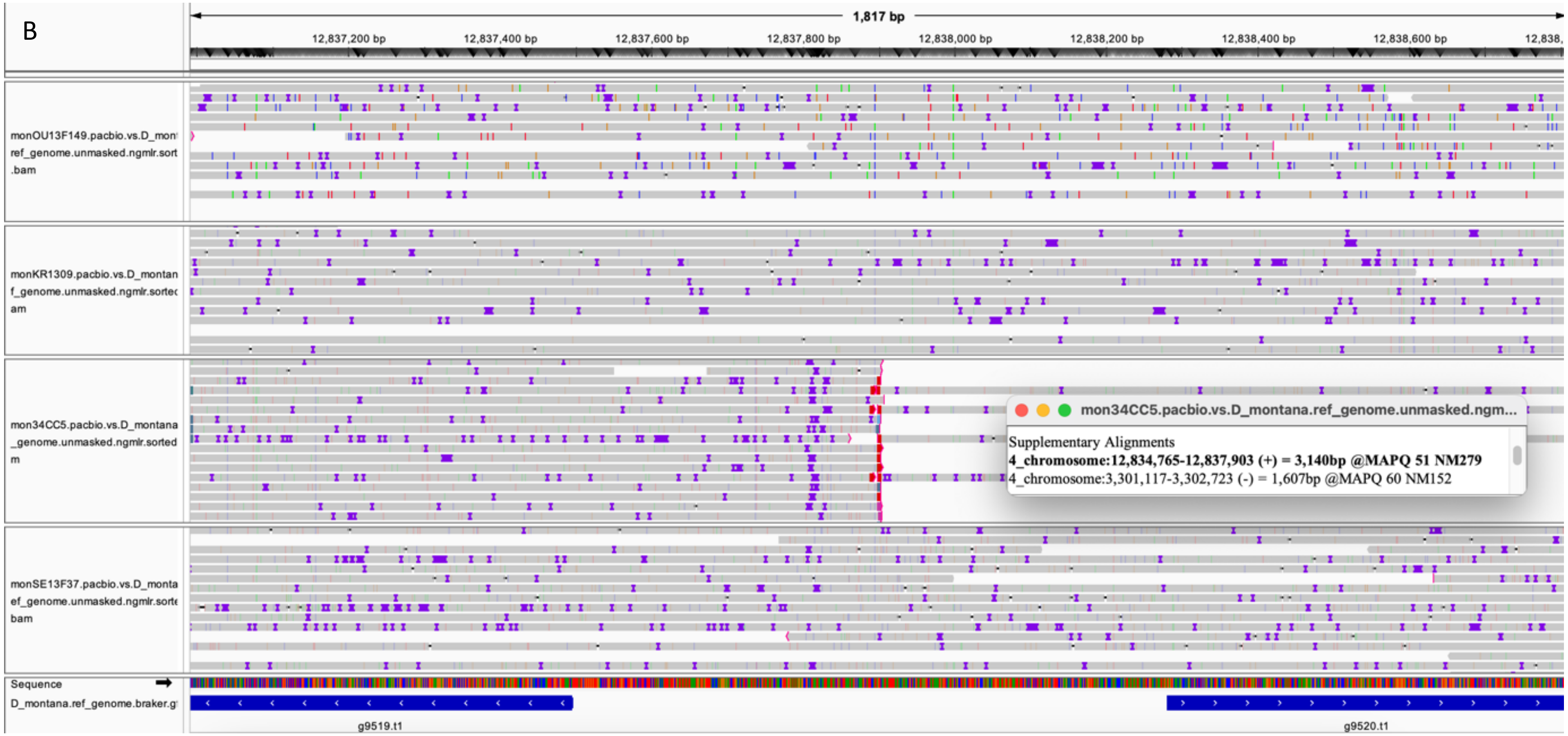
B
1,817 bp
12,837,200 bp
12,837,400 bp
12,837,600 bp
12,837,800 bp
12,838,000 bp
12,838,200 bp
12,838,400 bp
12,838,600 bp
monOU13F149.pacbio.vs.D_mon
ref_genome.unmasked.ngmlr.sort
.bam
monKR1309.pacbio.vs.D_montan
f_genome.unmasked.ngmlr.sortec
am
mon34CC5.pacbio.vs.D_montana
_genome.unmasked.ngmlr.sorted
m
mon34CC5.pacbio.vs.D_montana.ref_genome.unmasked.ngm...
Supplementary Alignments
4_chromosome:12,834,765-12,837,903 (+) = 3,140bp @MAPQ 51 NM279
4_chromosome:3,301,117-3,302,723 (-) = 1,607bp @MAPQ 60 NM152
monSE13F37.pacbio.vs.D_monta
ef_genome.unmasked.ngmlr.sorte
bam
Sequence
D_montana.ref_genome.braker.g
g9519.t1
g9520.t1

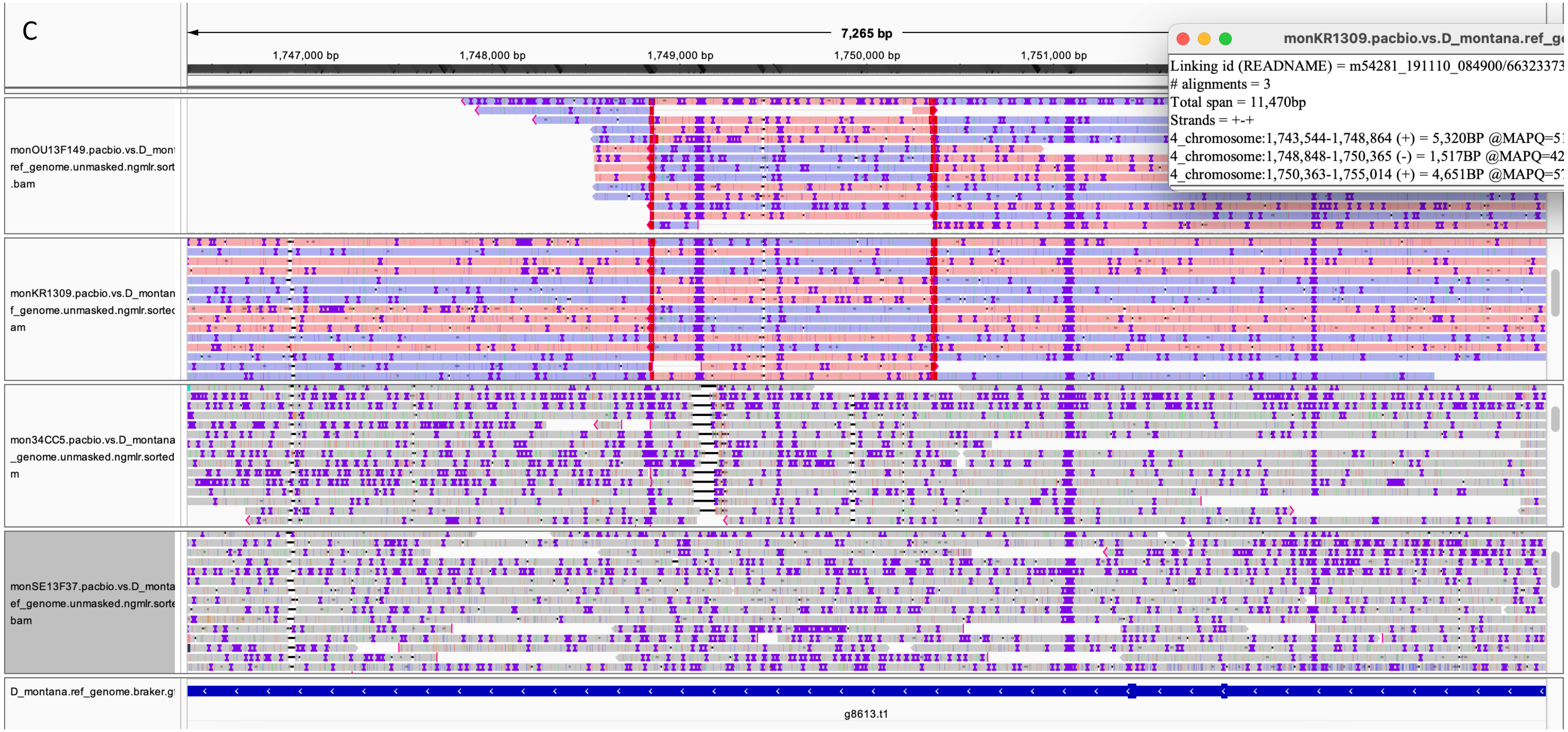

Figure S2. Inversion breakpoints illustrated with Integrative Genomics Viewer (IGV) v2.18.0 (Thorvaldsdottir et al. 2013). PacBio reads from different samples were mapped against the *D. montana* reference genome (Seward). (A) Proximal and (B) distal breakpoints of the large polymorphic inversion on chromosome 4. This inversion is fixed in the Crested Butte population (mon34CC5), as indicated by split reads (red tip of the read) and reversed orientation relative to the reference genome. (C) The small polymorphic inversion on chromosome 4, which is fixed in Fennoscandian and Asian populations (monOU13F149, monKR1309), shows split and inverted reads, with the inverted region shown in a different color compared to the rest of the reads. Gene IDs: g8672.t1 = tRNA-specific adenosine deaminase 1, g8673.t1 = UPF0428 protein CG16865, g9519.t1 = sorting nexin-17, g9520.t1 = tRNA-dihydrouridine(20a/20b) synthase [NAD(P)+]-like, g8613.t1 = leucine-rich repeat transmembrane neuronal protein 3.

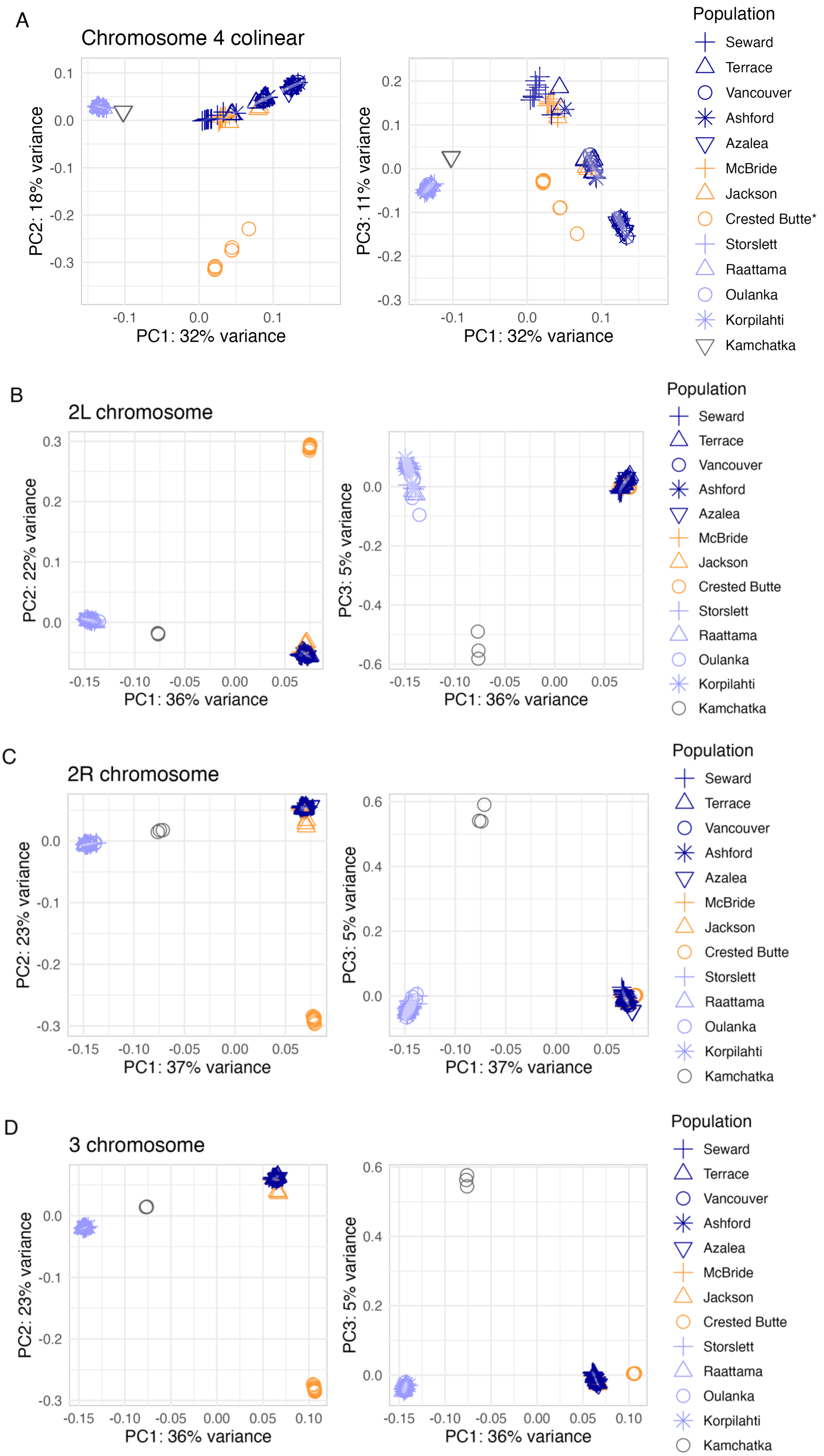

A
Chromosome 4 colinear
PC2: 18% variance
PC1: 32% variance
PC3: 11% variance
PC1: 32% variance
Population
Seward
Terrace
Vancouver
Ashford
Azalea
McBride
Jackson
Crested Butte*
Storslett
Raattama
Oulanka
Korpilahti
Kamchatka
B
2L chromosome
PC2: 22% variance
PC1: 36% variance
PC3: 5% variance
PC1: 36% variance
Population
Seward
Terrace
Vancouver
Ashford
Azalea
McBride
Jackson
Crested Butte
Storslett
Raattama
Oulanka
Korpilahti
Kamchatka
C
2R chromosome
PC2: 23% variance
PC1: 37% variance
PC3: 5% variance
PC1: 37% variance
Population
Seward
Terrace
Vancouver
Ashford
Azalea
McBride
Jackson
Crested Butte
Storslett
Raattama
Oulanka
Korpilahti
Kamchatka
D
3 chromosome
PC2: 23% variance
PC1: 36% variance
PC3: 5% variance
PC1: 36% variance
Population
Seward
Terrace
Vancouver
Ashford
Azalea
McBride
Jackson
Crested Butte
Storslett
Raattama
Oulanka
Korpilahti
Kamchatka

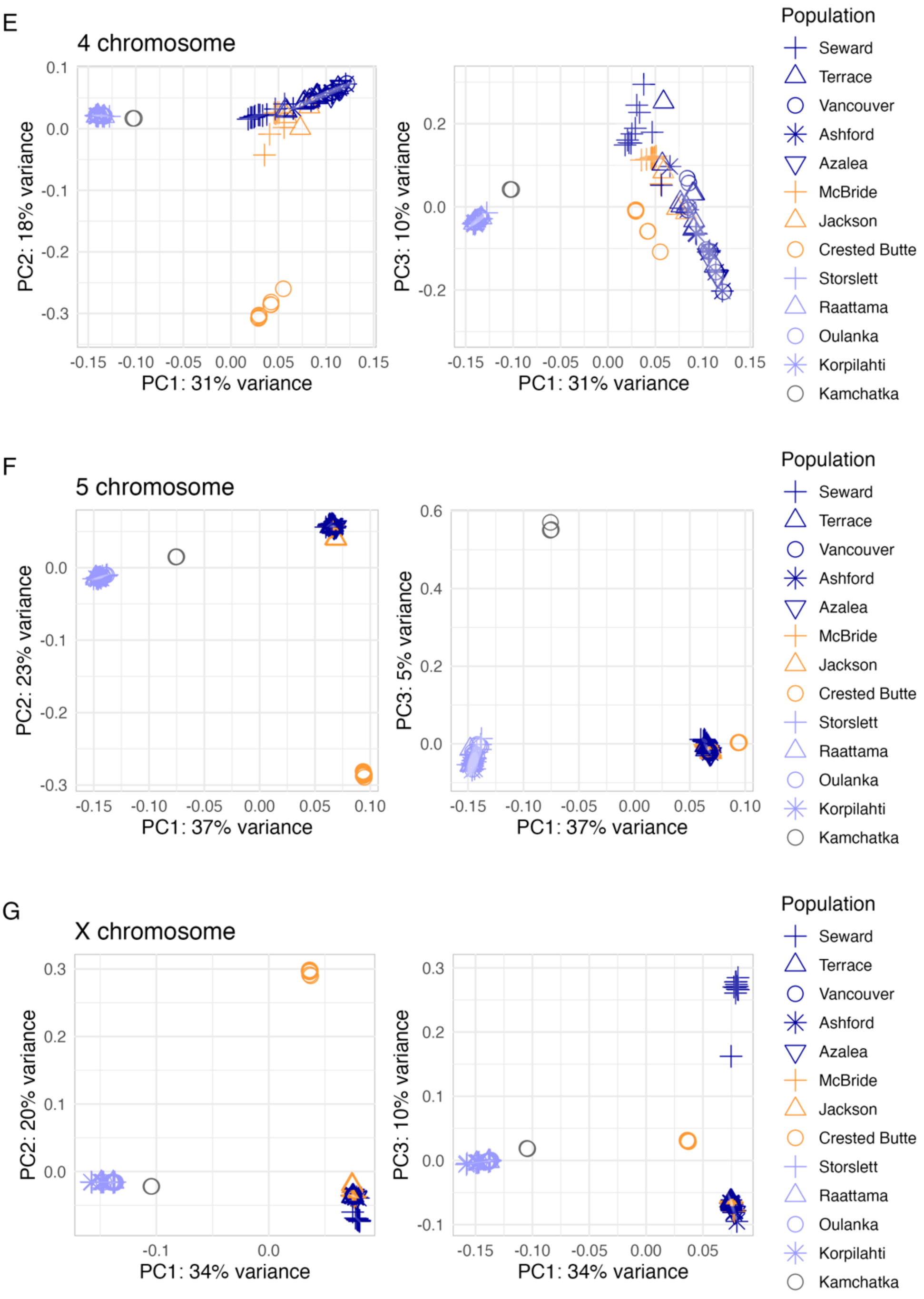

Figure S3. Genomic variation (SNPs from coding and non-coding regions) among *D. montana* populations (3-11 Illumina samples/population) summarized in a principal component analysis (PCA) for (A) colinear chromosome 4, (B) left arm of chromosome 2 (2L), (C) right arm of chromosome 2 (2R), (D) chromosome 3, (E) chromosome 4, (F) chromosome 5, and (G) the X chromosome. See details in Table S13.

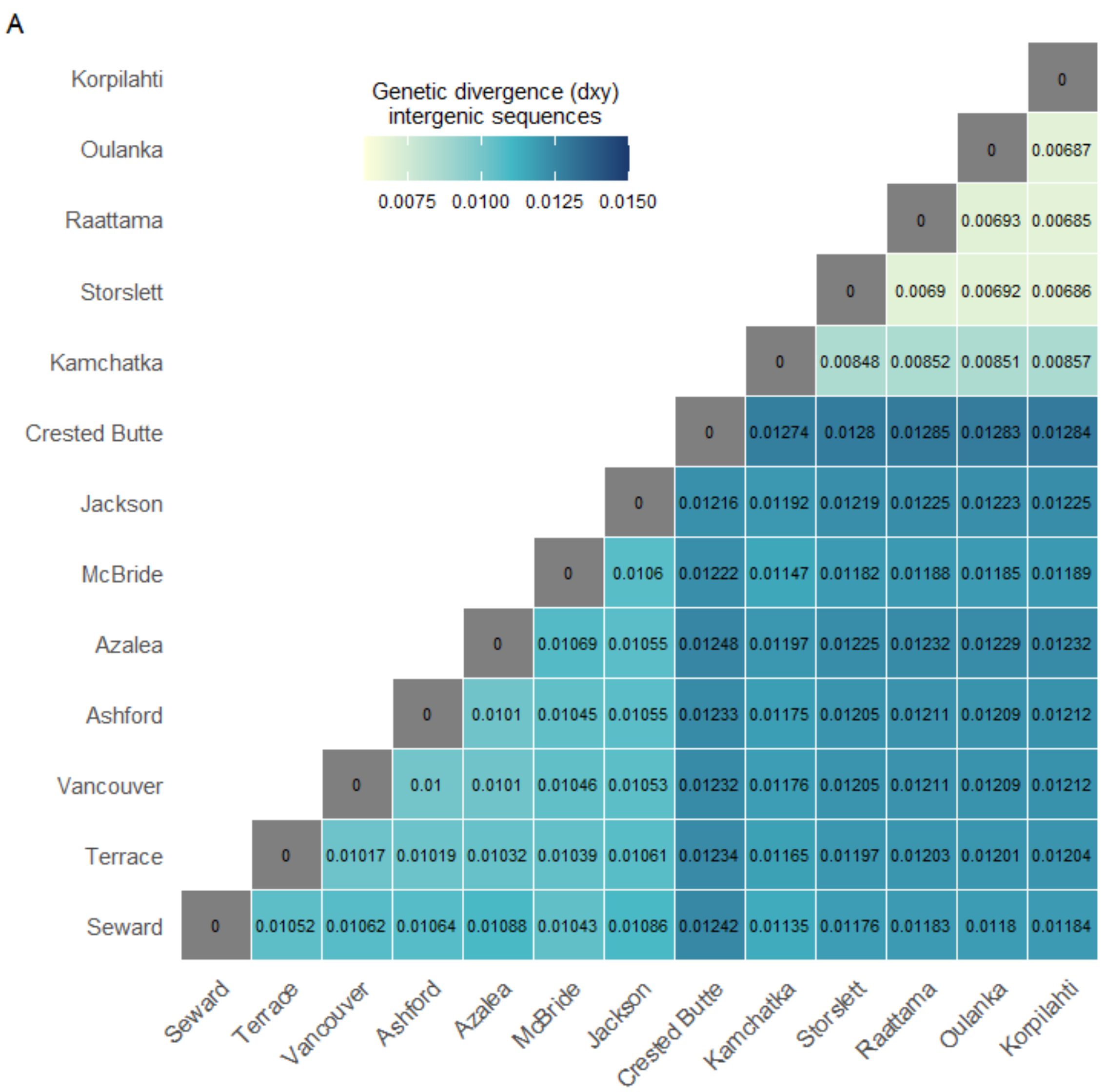
A
Genetic divergence (dxy)
intergenic sequences
0.0075 0.0100 0.0125 0.0150
Korpilahti 0
Oulanka 0 0.00687
Raattama 0 0.00693 0.00685
Storslett 0 0.0069 0.00692 0.00686
Kamchatka 0 0.00848 0.00852 0.00851 0.00857
Crested Butte 0 0.01274 0.0128 0.01285 0.01283 0.01284
Jackson 0 0.01216 0.01192 0.01219 0.01225 0.01223 0.01225
McBride 0 0.0106 0.01222 0.01147 0.01182 0.01188 0.01185 0.01189
Azalea 0 0.01069 0.01055 0.01248 0.01197 0.01225 0.01232 0.01229 0.01232
Ashford 0 0.0101 0.01045 0.01055 0.01233 0.01175 0.01205 0.01211 0.01209 0.01212
Vancouver 0 0.01 0.0101 0.01046 0.01053 0.01232 0.01176 0.01205 0.01211 0.01209 0.01212
Terrace 0 0.01017 0.01019 0.01032 0.01039 0.01061 0.01234 0.01165 0.01197 0.01203 0.01201 0.01204
Seward 0 0.01052 0.01062 0.01064 0.01088 0.01043 0.01086 0.01242 0.01135 0.01176 0.01183 0.0118 0.01184
Seward Terrace Vancouver Ashford Azalea McBride Jackson Crested Butte Kamchatka Storslett Raattama Oulanka Korpilahti

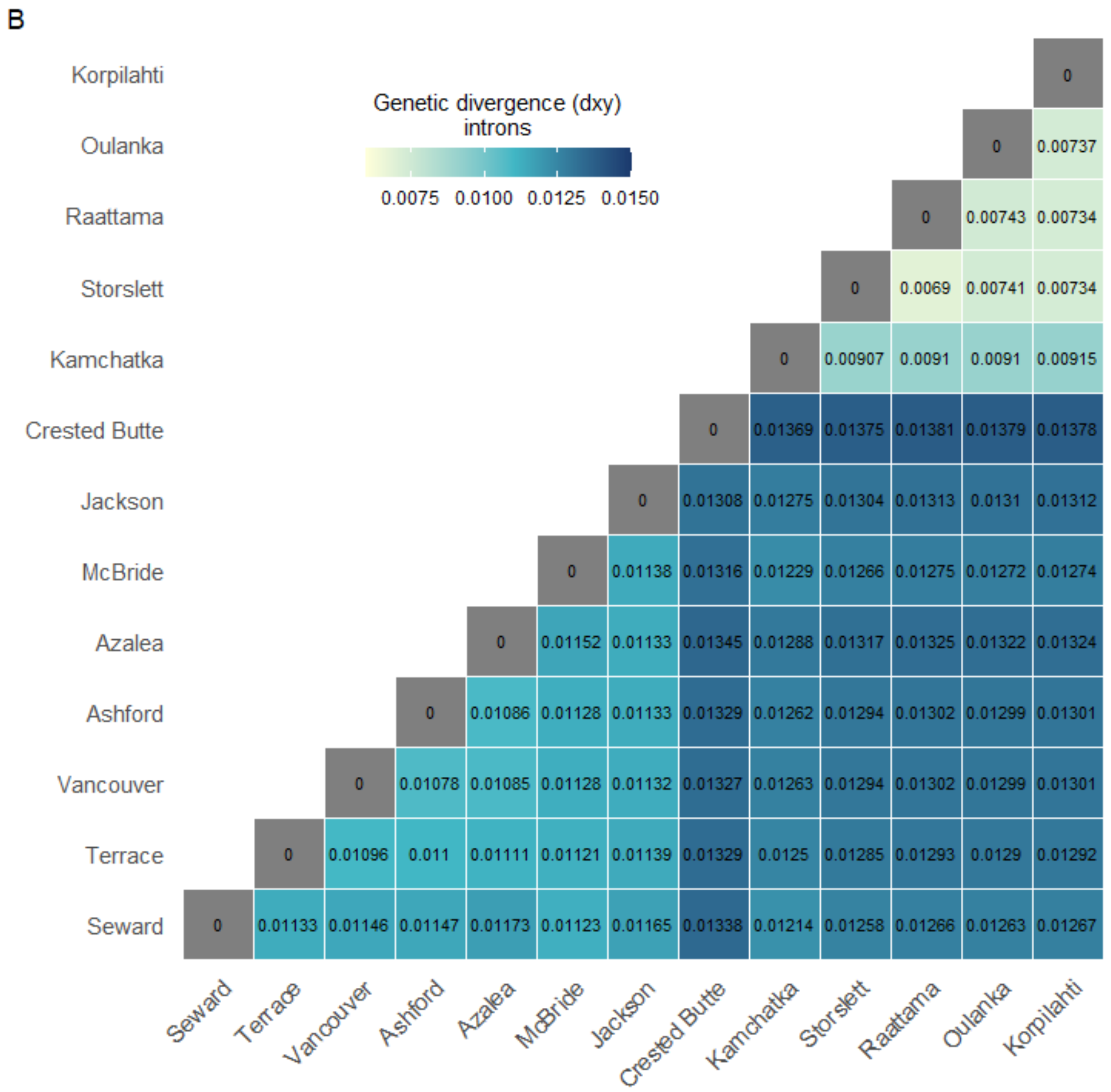
B
Genetic divergence (dxy)
introns
0.0075 0.0100 0.0125 0.0150
Korpilahti 0
Oulanka 0 0.00737
Raattama 0 0.00743 0.00734
Storslett 0 0.0069 0.00741 0.00734
Kamchatka 0 0.00907 0.0091 0.0091 0.00915
Crested Butte 0 0.01369 0.01375 0.01381 0.01379 0.01378
Jackson 0 0.01308 0.01275 0.01304 0.01313 0.0131 0.01312
McBride 0 0.01138 0.01316 0.01229 0.01266 0.01275 0.01272 0.01274
Azalea 0 0.01152 0.01133 0.01345 0.01288 0.01317 0.01325 0.01322 0.01324
Ashford 0 0.01086 0.01128 0.01133 0.01329 0.01262 0.01294 0.01302 0.01299 0.01301
Vancouver 0 0.01078 0.01085 0.01128 0.01132 0.01327 0.01263 0.01294 0.01302 0.01299 0.01301
Terrace 0 0.01096 0.011 0.01111 0.01121 0.01139 0.01329 0.0125 0.01285 0.01293 0.0129 0.01292
Seward 0 0.01133 0.01146 0.01147 0.01173 0.01123 0.01165 0.01338 0.01214 0.01258 0.01266 0.01263 0.01267
Seward Terrace Vancouver Ashford Azalea McBride Jackson Crested Butte Kamchatka Storslett Raattama Oulanka Korpilahti

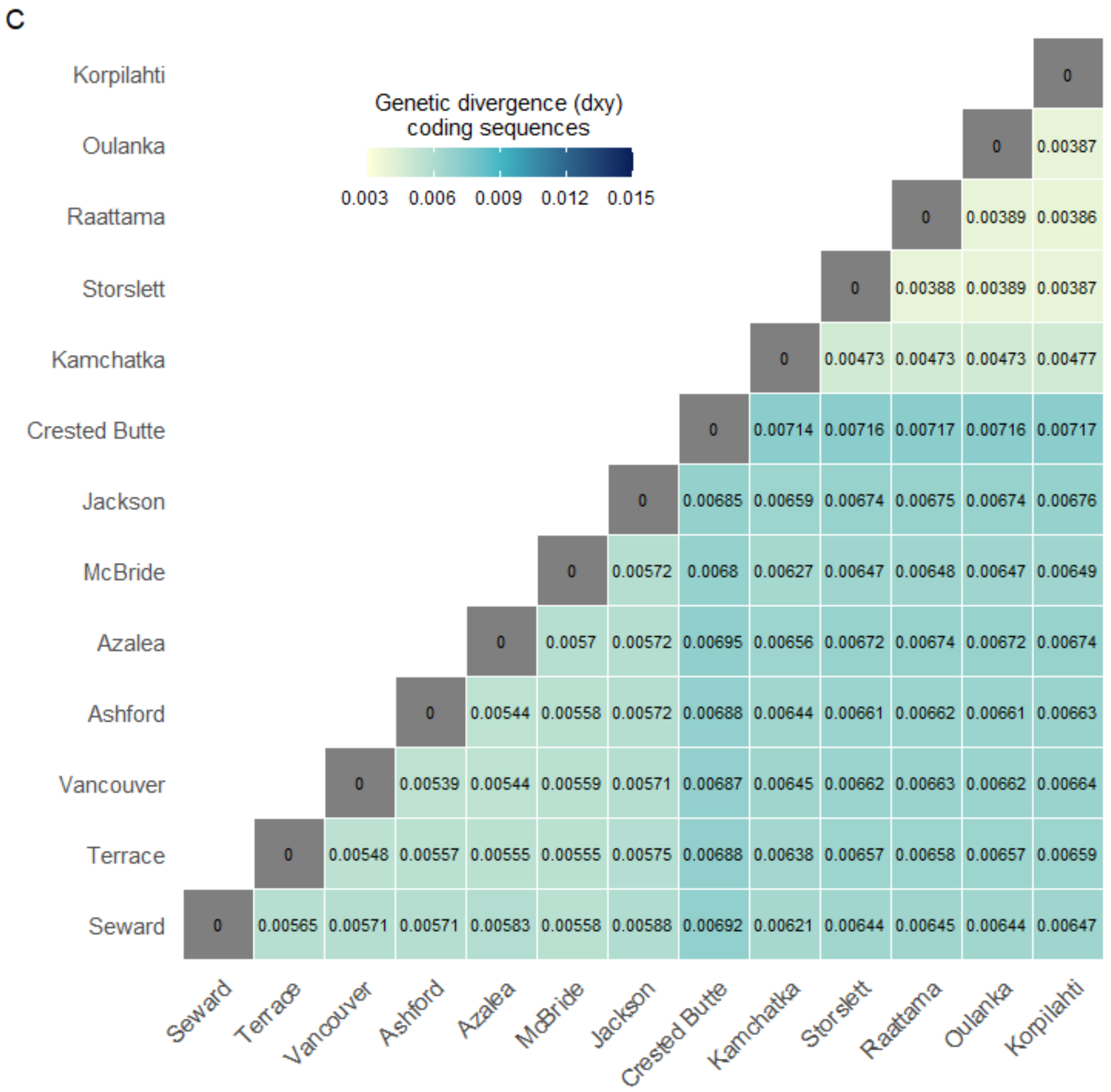

Figure S4. Mean genetic divergence (d_{xy}) of all population pairs for (A) intergenic, (B), intronic and (C) coding sequences.

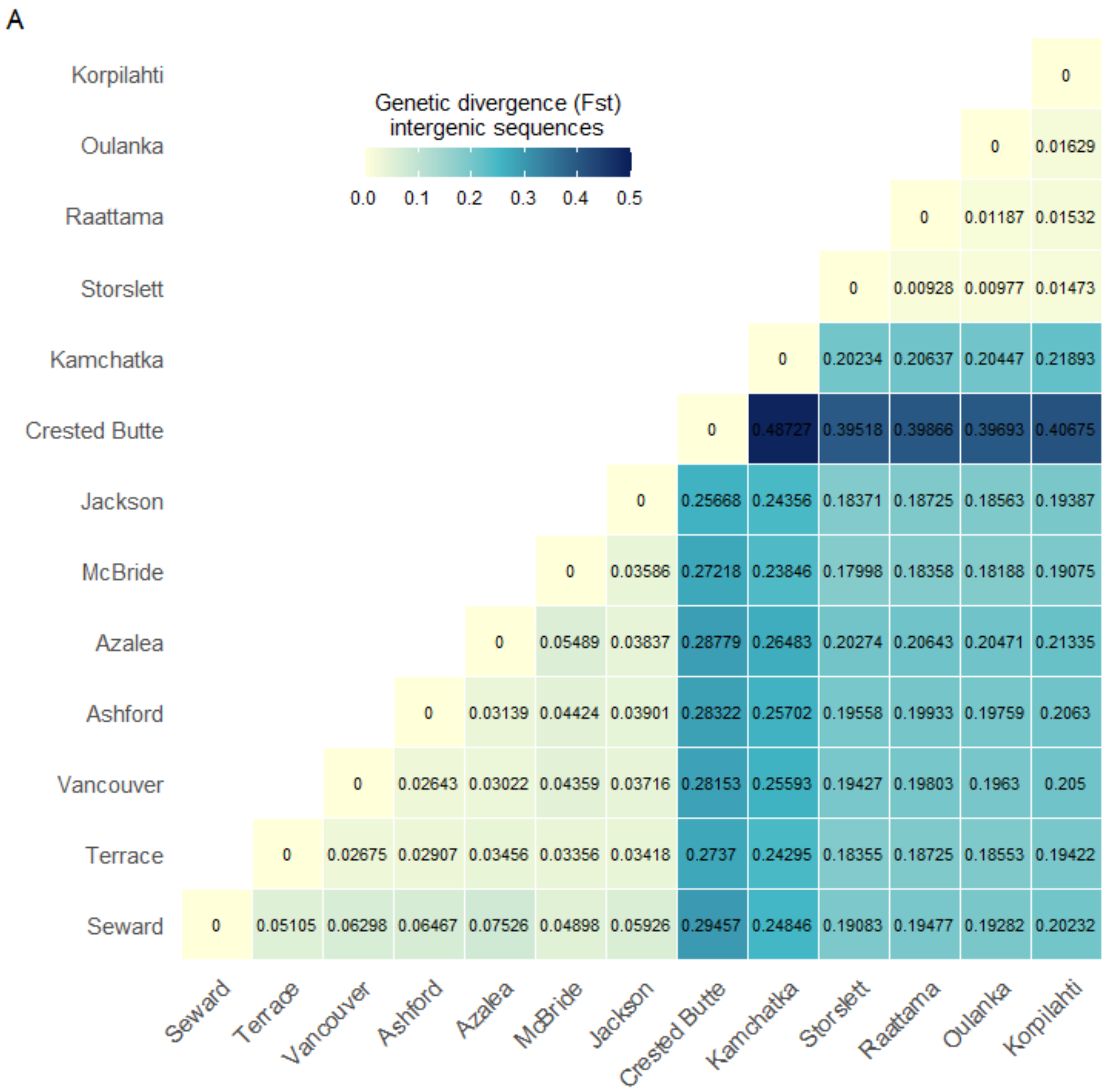
A
Genetic divergence (Fst)
intergenic sequences
0.0 0.1 0.2 0.3 0.4 0.5
Korpilahti 0
Oulanka 0 0.01629
Raattama 0 0.01187 0.01532
Storslett 0 0.00928 0.00977 0.01473
Kamchatka 0 0.20234 0.20637 0.20447 0.21893
Crested Butte 0 0.48727 0.39518 0.39866 0.39693 0.40675
Jackson 0 0.25668 0.24356 0.18371 0.18725 0.18563 0.19387
McBride 0 0.03586 0.27218 0.23846 0.17998 0.18358 0.18188 0.19075
Azalea 0 0.05489 0.03837 0.28779 0.26483 0.20274 0.20643 0.20471 0.21335
Ashford 0 0.03139 0.04424 0.03901 0.28322 0.25702 0.19558 0.19933 0.19759 0.2063
Vancouver 0 0.02643 0.03022 0.04359 0.03716 0.28153 0.25593 0.19427 0.19803 0.1963 0.205
Terrace 0 0.02675 0.02907 0.03456 0.03356 0.03418 0.2737 0.24295 0.18355 0.18725 0.18553 0.19422
Seward 0 0.05105 0.06298 0.06467 0.07526 0.04898 0.05926 0.29457 0.24846 0.19083 0.19477 0.19282 0.20232
Seward Terrace Vancouver Ashford Azalea McBride Jackson Crested Butte Kamchatka Storslett Raattama Oulanka Korpilahti

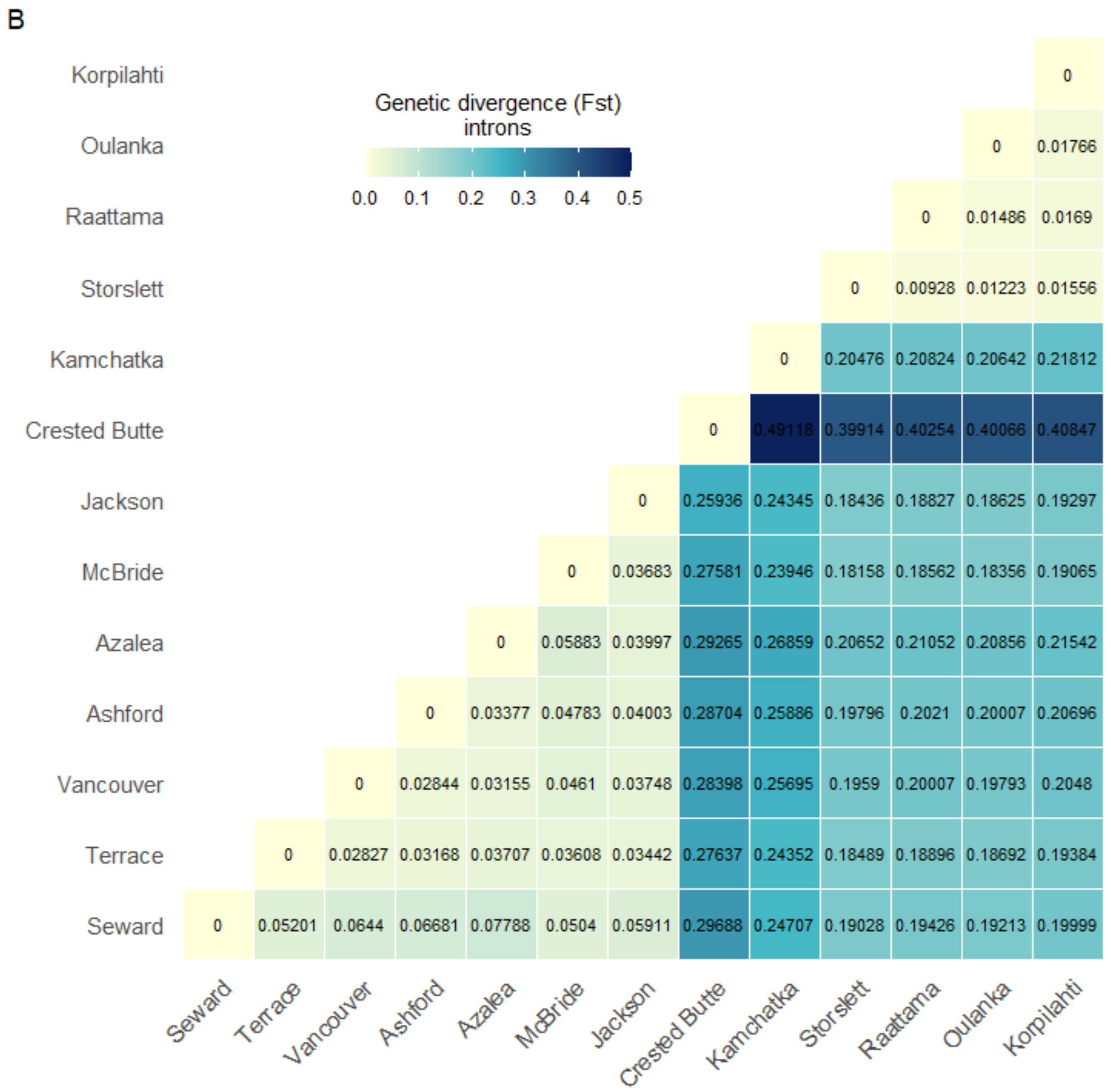
B
Genetic divergence (Fst)
introns
0.0 0.1 0.2 0.3 0.4 0.5
Korpilahti 0
Oulanka 0 0.01766
Raattama 0 0.01486 0.0169
Storslett 0 0.00928 0.01223 0.01556
Kamchatka 0 0.20476 0.20824 0.20642 0.21812
Crested Butte 0 0.49118 0.39914 0.40254 0.40066 0.40847
Jackson 0 0.25936 0.24345 0.18436 0.18827 0.18625 0.19297
McBride 0 0.03683 0.27581 0.23946 0.18158 0.18562 0.18356 0.19065
Azalea 0 0.05883 0.03997 0.29265 0.26859 0.20652 0.21052 0.20856 0.21542
Ashford 0 0.03377 0.04783 0.04003 0.28704 0.25886 0.19796 0.2021 0.20007 0.20696
Vancouver 0 0.02844 0.03155 0.0461 0.03748 0.28398 0.25695 0.1959 0.20007 0.19793 0.2048
Terrace 0 0.02827 0.03168 0.03707 0.03608 0.03442 0.27637 0.24352 0.18489 0.18896 0.18692 0.19384
Seward 0 0.05201 0.0644 0.06681 0.07788 0.0504 0.05911 0.29688 0.24707 0.19028 0.19426 0.19213 0.19999
Seward Terrace Vancouver Ashford Azalea McBride Jackson Crested Butte Kamchatka Storslett Raattama Oulanka Korpilahti

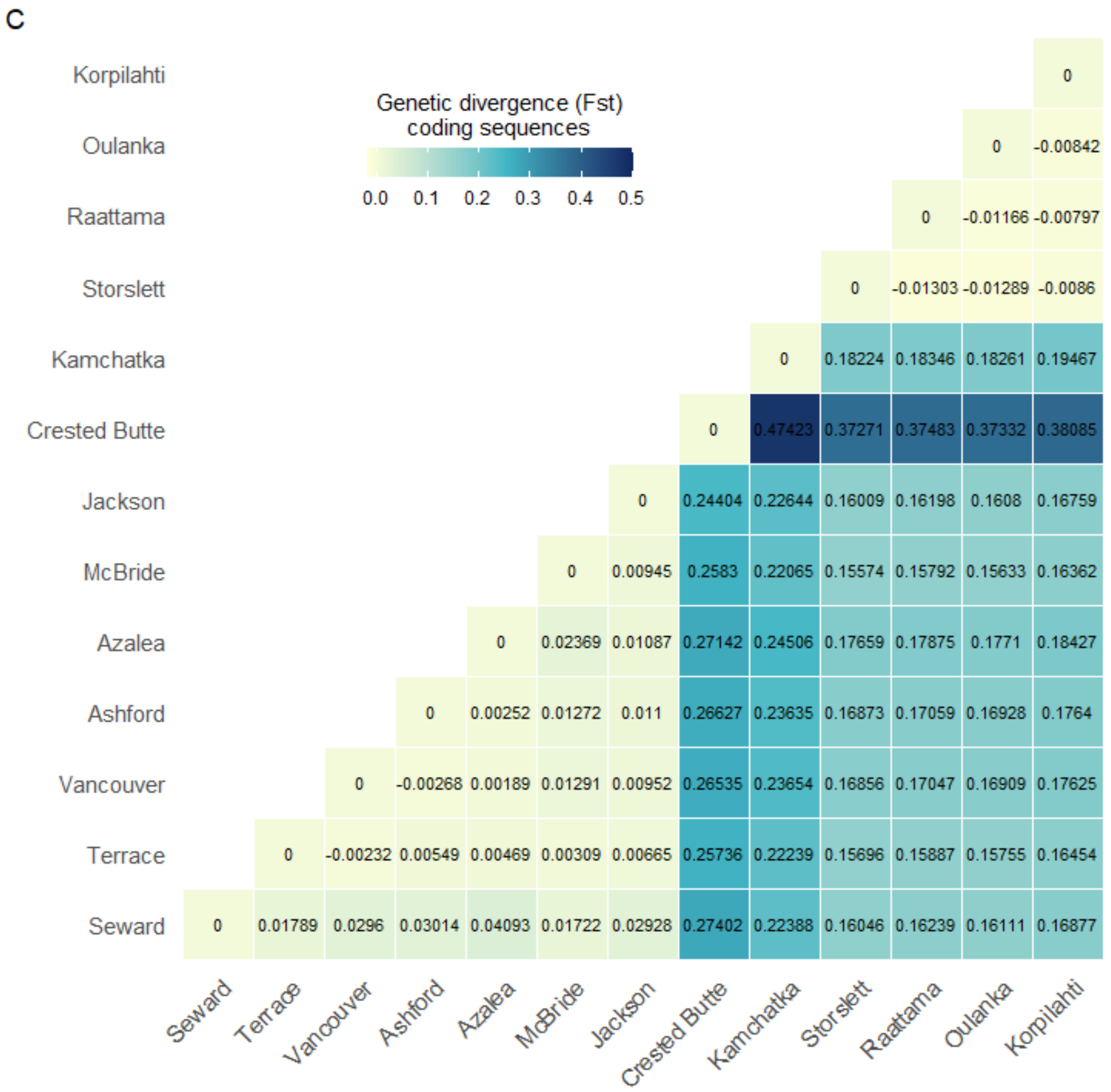

Figure S5. Mean genetic divergence (F_{st}) of all population pairs for (A) intergenic, (B), intronic and (C) coding sequences.

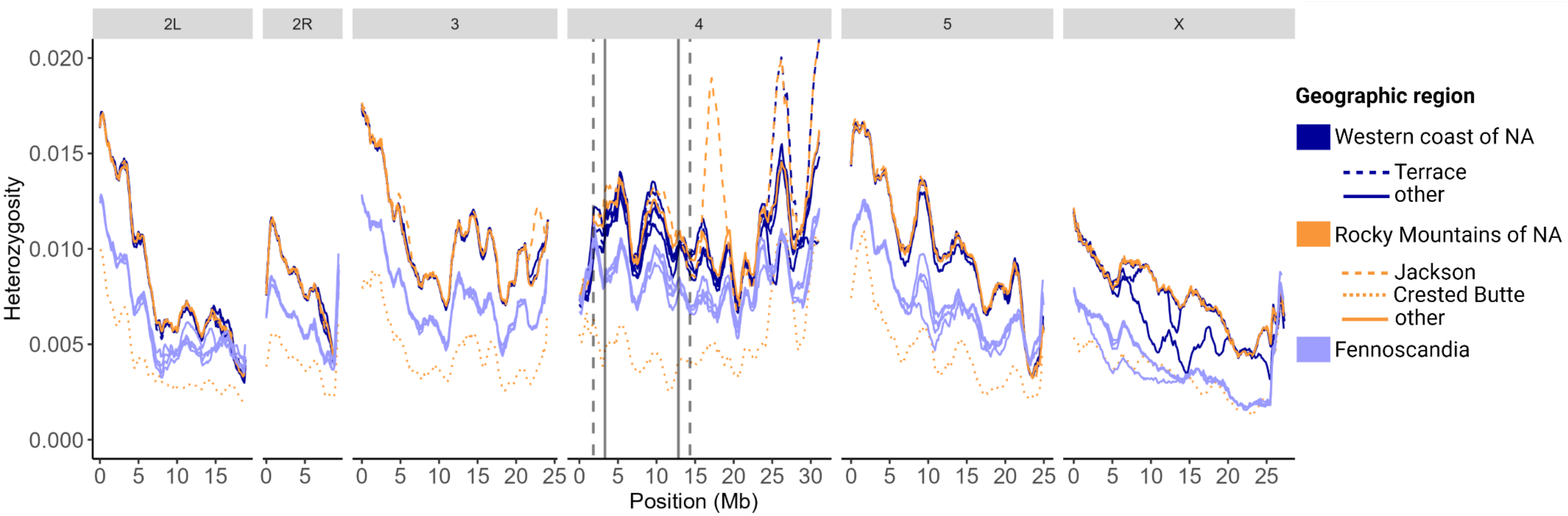

Figure S6. Genome-wide heterozygosity (*H*) of *D. montana* populations originating from the western coast and Rocky Mountains of North America (NA), and Fennoscandia. Populations are highlighted with dashed lines whenever their genetic diversity strongly deviates from that of the other populations of the same geographic region. The inversion breakpoints are marked with vertical solid lines. Since recombination is often suppressed beyond the inverted region into the colinear region, 1.5Mb buffer region was added at both ends of the inversion and indicated with vertical dashed lines.

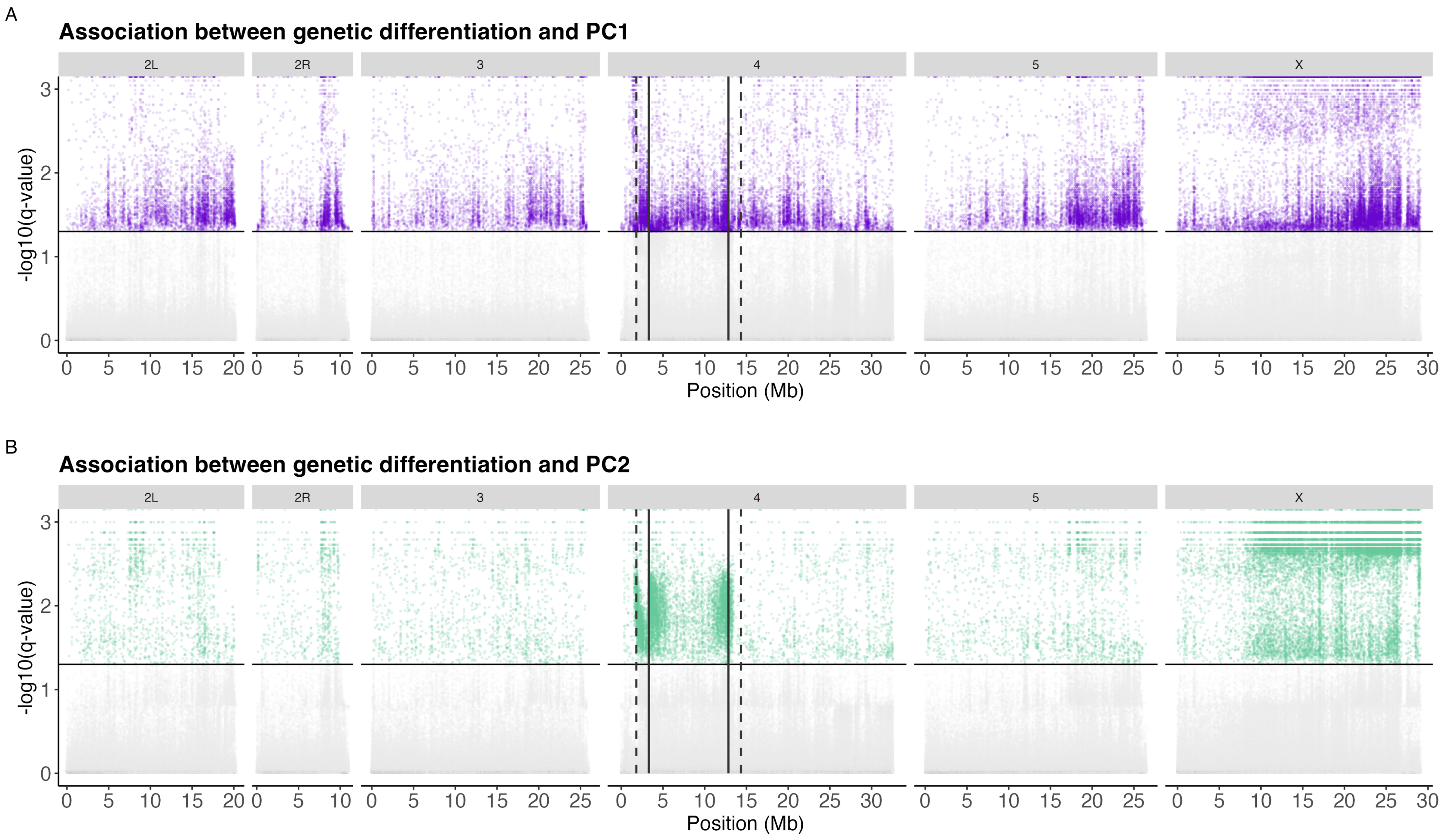

A
Association between genetic differentiation and PC1
2L
2R
3
4
5
X
-log10(q-value)
0
1
2
3
0 5 10 15 20
0 5 10
0 5 10 15 20 25
0 5 10 15 20 25 30
0 5 10 15 20 25
0 5 10 15 20 25 30
Position (Mb)
B
Association between genetic differentiation and PC2
2L
2R
3
4
5
X
-log10(q-value)
Position (Mb)

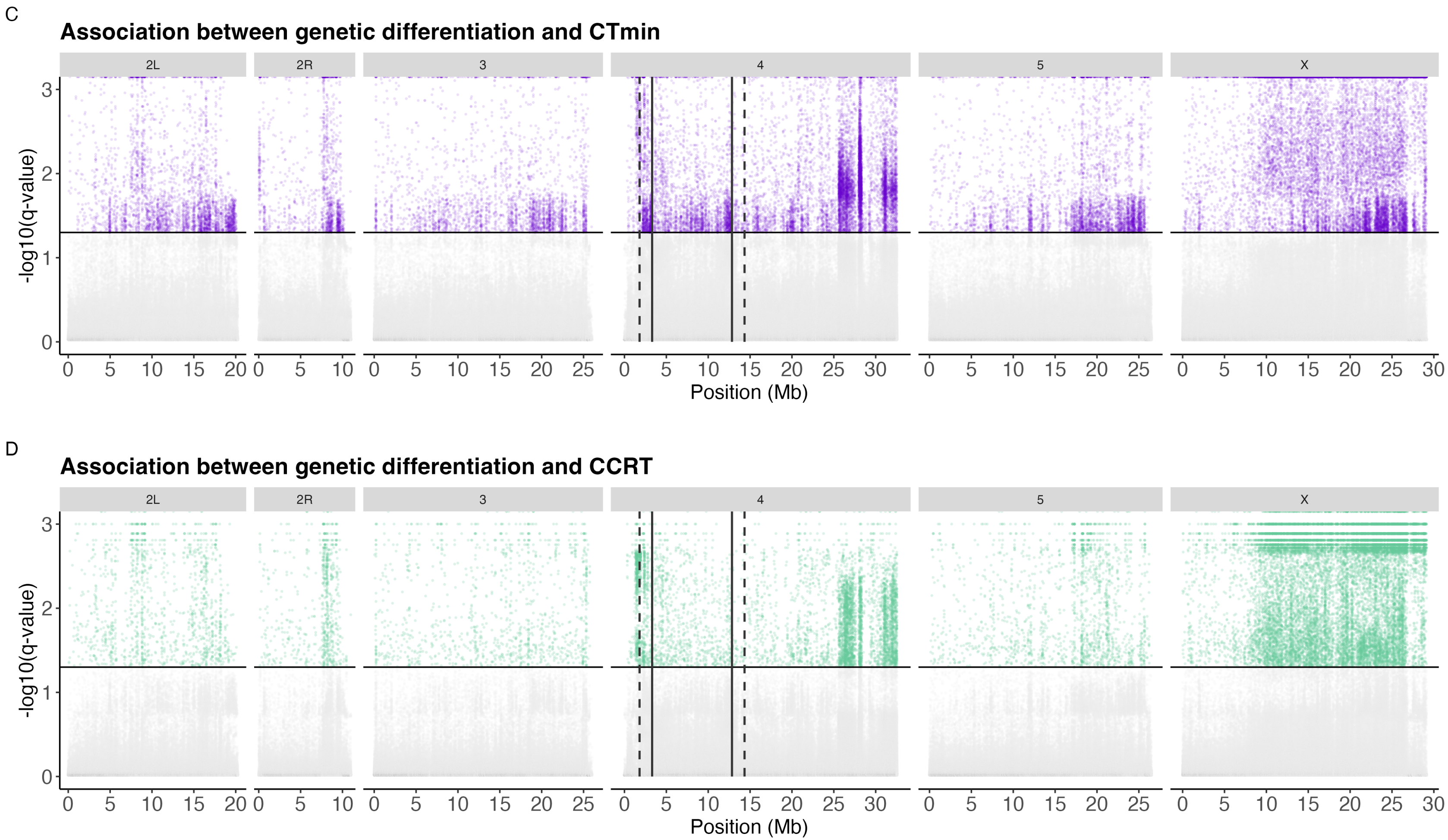

Figure S7. Genotype-environment and genotype-phenotype association analysis (BayeScEnv). Points above the horizontal line indicate significant SNPs, describing the association between genetic differentiation and (A) PC1, (B) PC2, (C) CT_{min} and (D) CCRT. q-values for the g parameter of two chains (Fig. S8-S11) were used to control the false positive rate at 0.05, i.e. SNPs with q-values>1.3, to obtain the final candidate SNPs. The solid vertical lines represent the inversion breakpoints, while the dashed vertical lines mark a conservative estimate of 1.5Mb beyond the breakpoints, where recombination is expected to remain reduced.

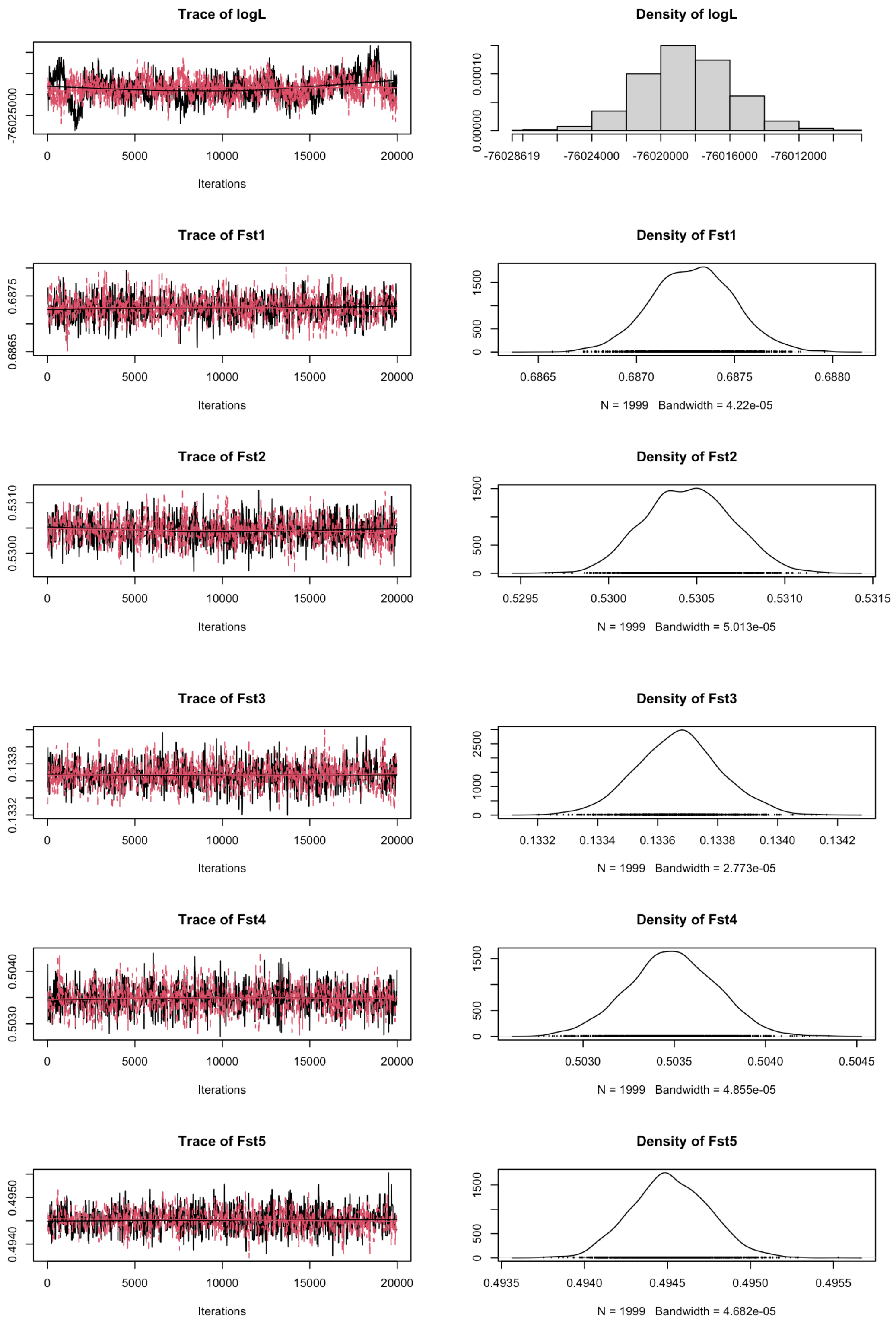

Trace of logL
-76025000
0
5000
10000
15000
20000
Iterations
Density of logL
0.00000
0.00010
-76028619
-76024000
-76020000
-76016000
-76012000
Trace of Fst1
0.6865
0.6875
Iterations
Density of Fst1
0
500
1500
0.6865
0.6870
0.6875
0.6880
N = 1999 Bandwidth = 4.22e-05
Trace of Fst2
0.5300
0.5310
Iterations
Density of Fst2
0.5295
0.5300
0.5305
0.5310
0.5315
N = 1999 Bandwidth = 5.013e-05
Trace of Fst3
0.1332
0.1338
Iterations
Density of Fst3
1000
2500
0.1332
0.1334
0.1336
0.1338
0.1340
0.1342
N = 1999 Bandwidth = 2.773e-05
Trace of Fst4
0.5030
0.5040
Iterations
Density of Fst4
0.5030
0.5035
0.5040
0.5045
N = 1999 Bandwidth = 4.855e-05
Trace of Fst5
0.4940
0.4950
Iterations
Density of Fst5
0.4935
0.4940
0.4945
0.4950
0.4955
N = 1999 Bandwidth = 4.682e-05

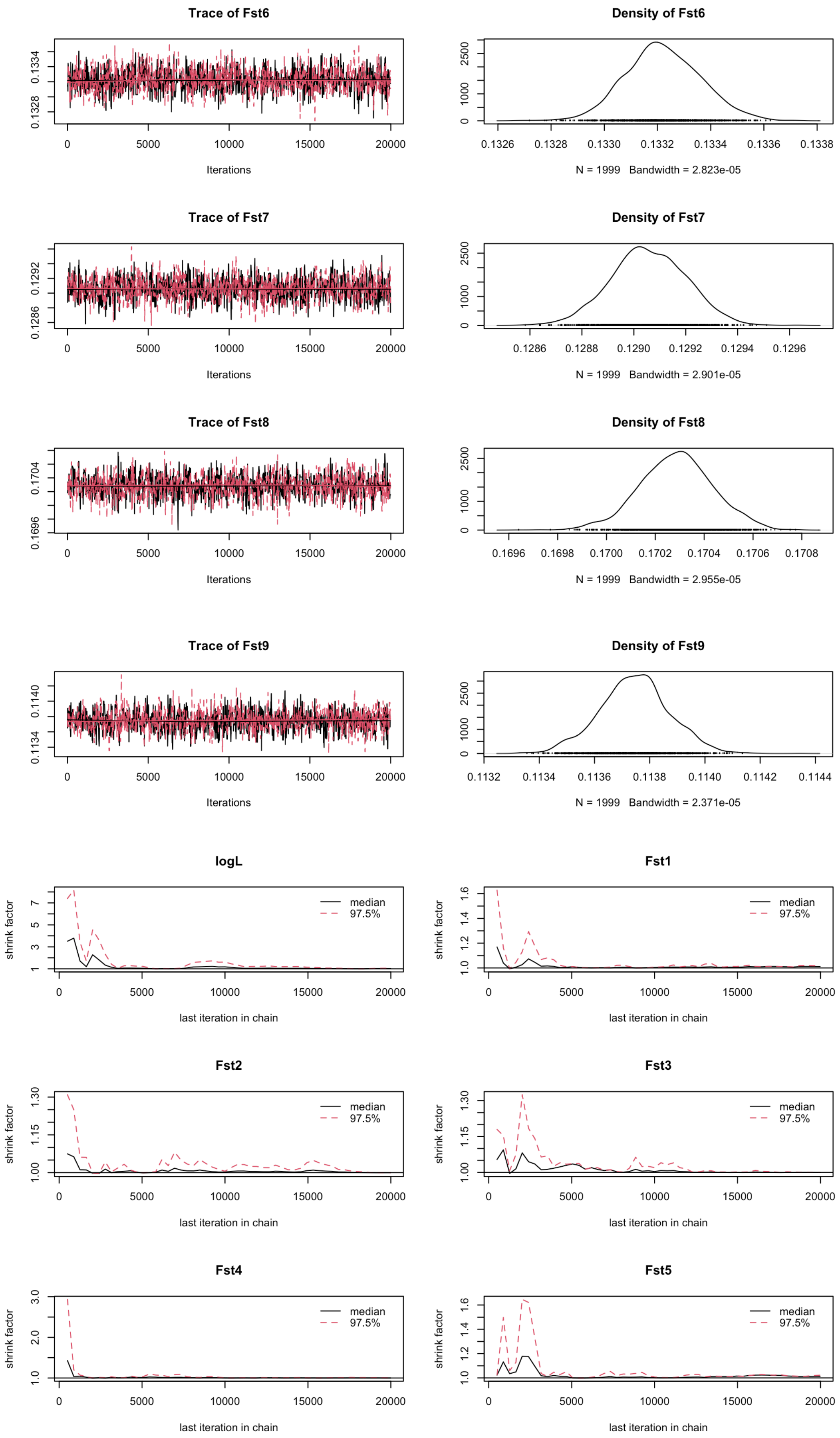
Trace of Fst6
Iterations
Density of Fst6
N = 1999 Bandwidth = 2.823e-05
Trace of Fst7
Iterations
Density of Fst7
N = 1999 Bandwidth = 2.901e-05
Trace of Fst8
Iterations
Density of Fst8
N = 1999 Bandwidth = 2.955e-05
Trace of Fst9
Iterations
Density of Fst9
N = 1999 Bandwidth = 2.371e-05
logL
Fst1
Fst2
Fst3
Fst4
Fst5
shrink factor
last iteration in chain
median
97.5%

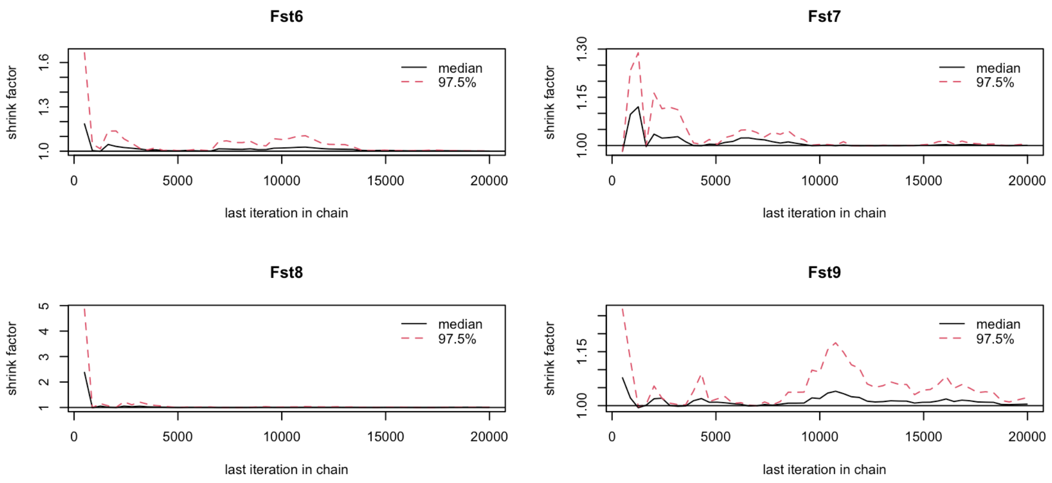

Figure S8. Trace and diagnostic Gelman plots for BayeScEnv MCMC chains with PC1 as the environmental variable. Trace plots show parameter values across iterations of MCMC chains. Gelman plots show the Gelman-Rubin shrink factors that summarise variation in parameter estimates across the two chains that were run.

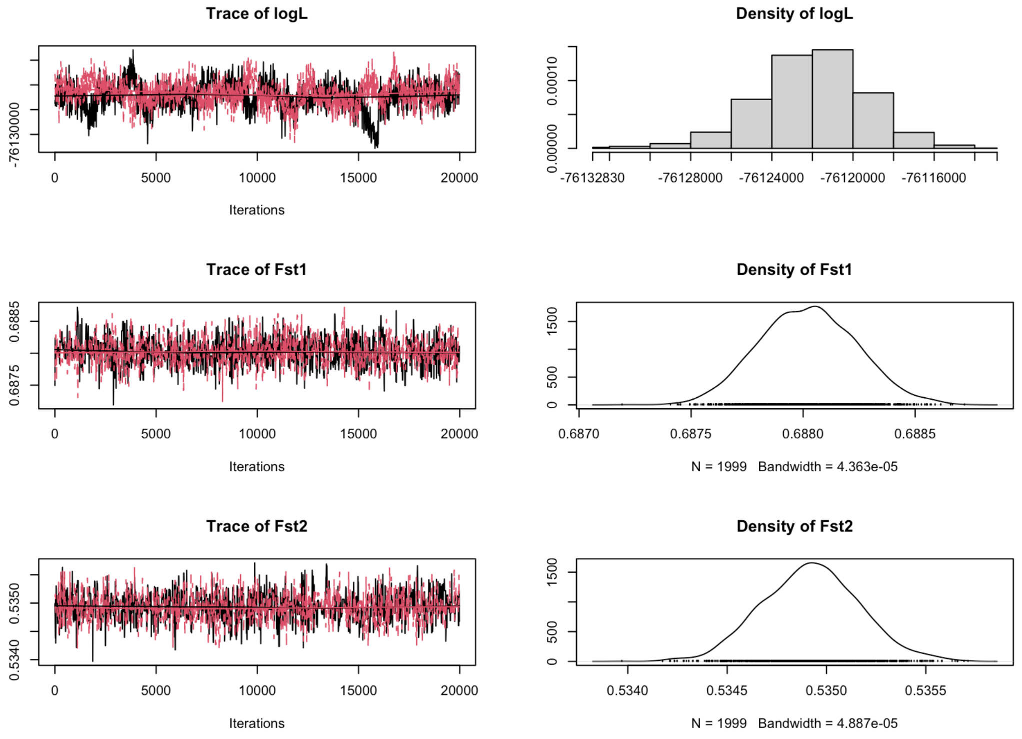

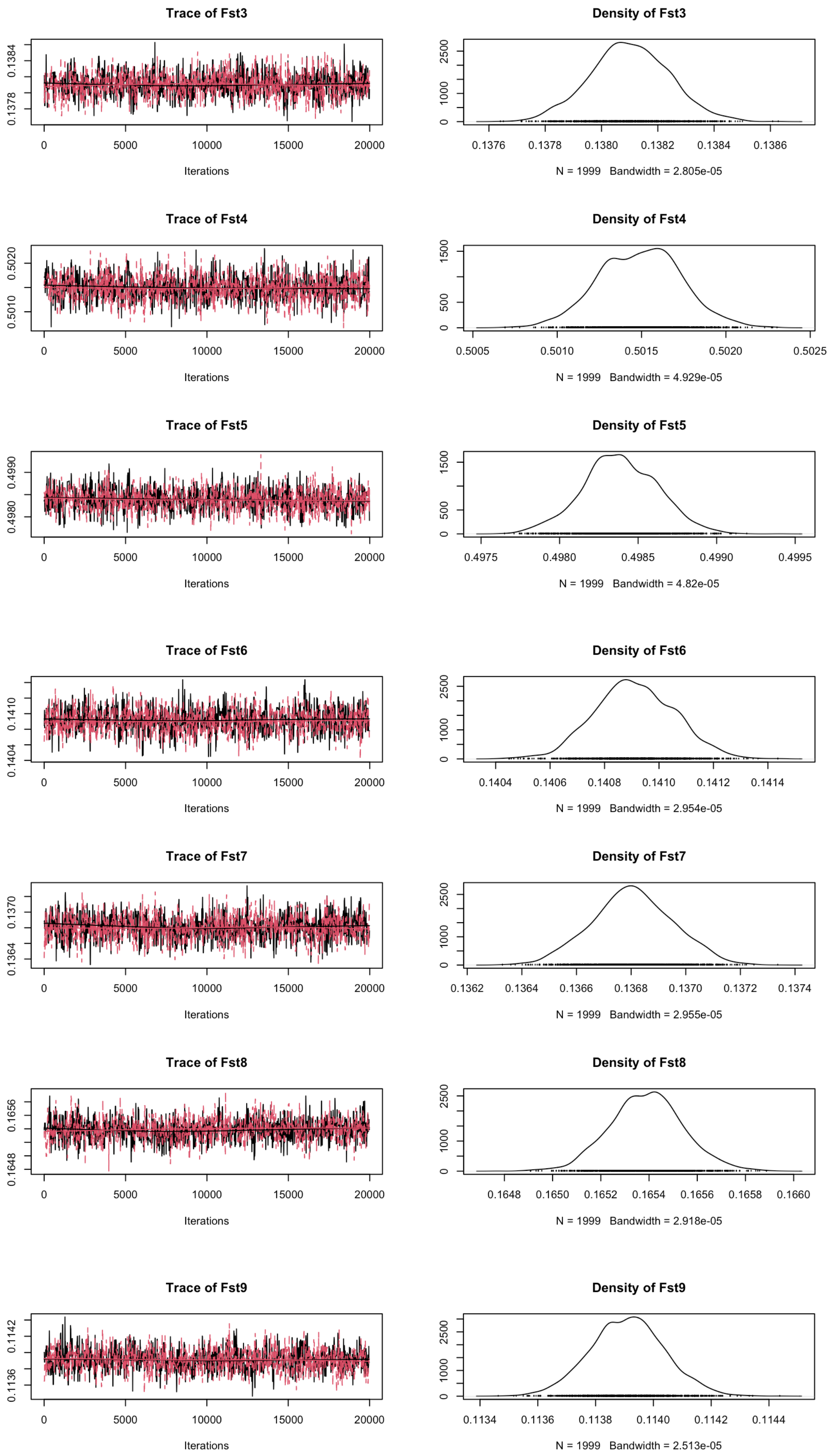
Trace of Fst3
Density of Fst3
Iterations
N = 1999 Bandwidth = 2.805e-05
Trace of Fst4
Density of Fst4
Iterations
N = 1999 Bandwidth = 4.929e-05
Trace of Fst5
Density of Fst5
Iterations
N = 1999 Bandwidth = 4.82e-05
Trace of Fst6
Density of Fst6
Iterations
N = 1999 Bandwidth = 2.954e-05
Trace of Fst7
Density of Fst7
Iterations
N = 1999 Bandwidth = 2.955e-05
Trace of Fst8
Density of Fst8
Iterations
N = 1999 Bandwidth = 2.918e-05
Trace of Fst9
Density of Fst9
Iterations
N = 1999 Bandwidth = 2.513e-05

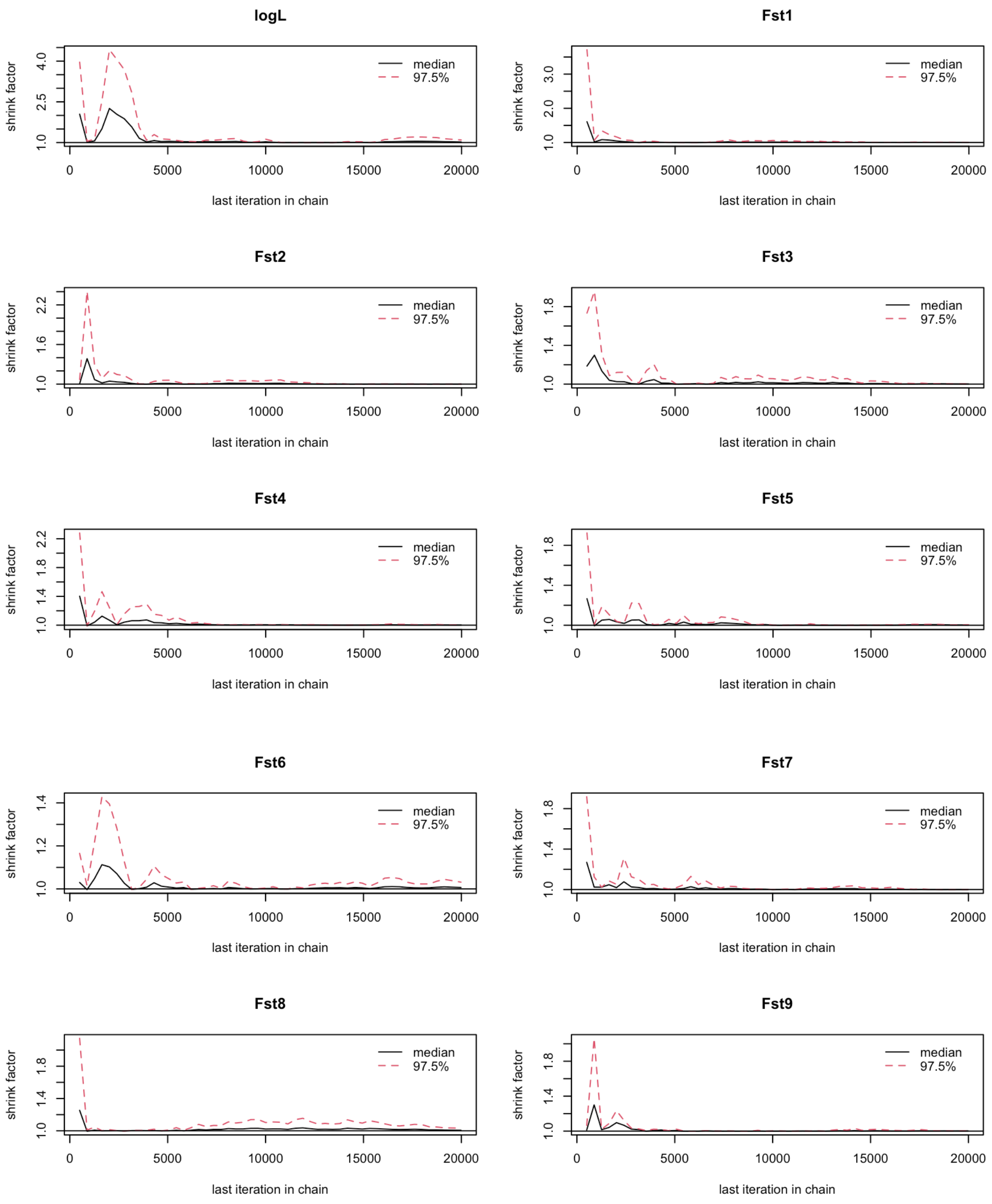

Figure S9. Trace and diagnostic Gelman plots for BayeScEnv MCMC chains with PC2 as the environmental variable. Trace plots show parameter values across iterations of MCMC chains. Gelman plots show the Gelman-Rubin shrink factors that summarise variation in parameter estimates across the two chains that were run.

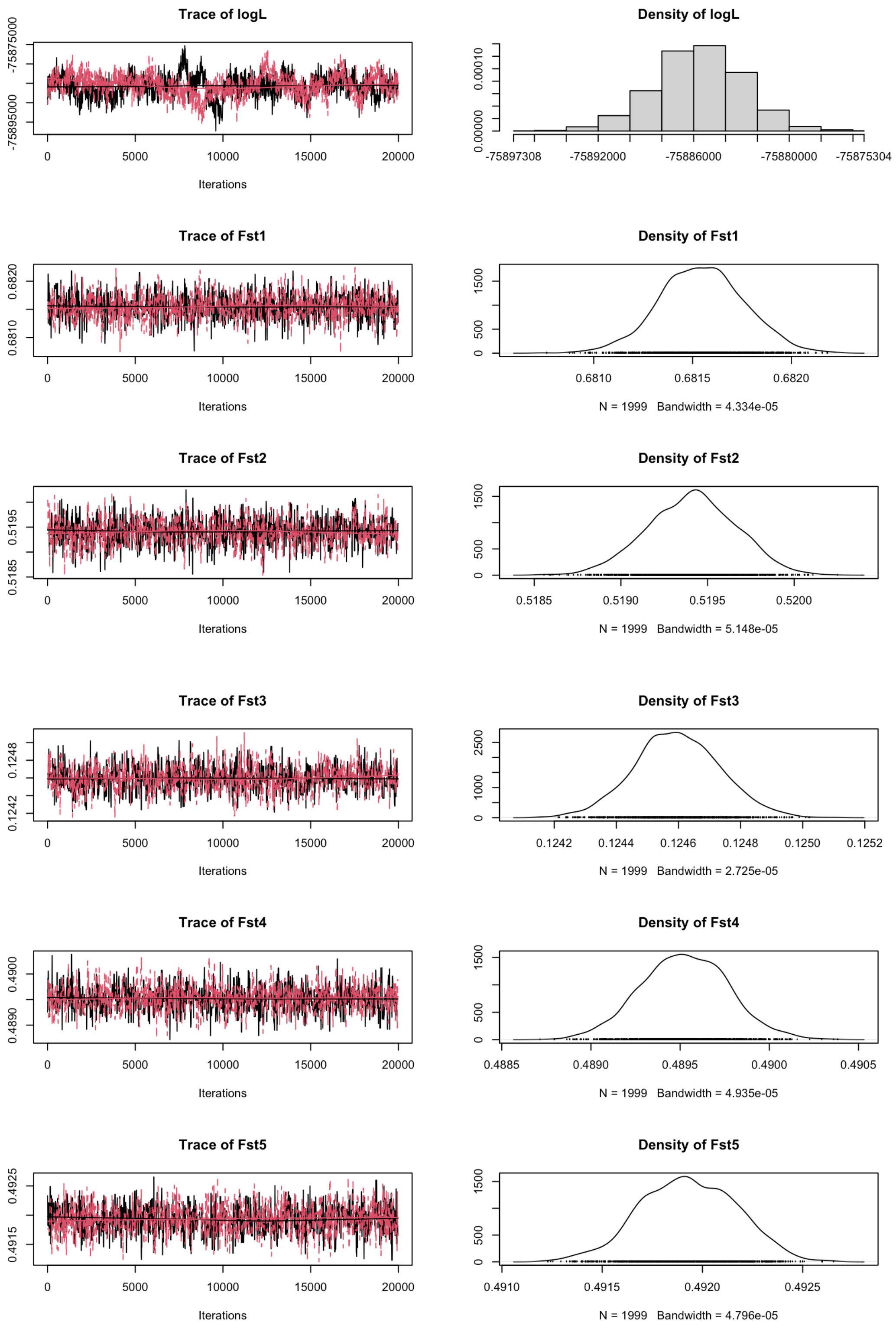
Trace of logL
-75875000
-75895000
0
5000
10000
15000
20000
Iterations
Density of logL
0.00010
0.00000
-75897308
-75892000
-75886000
-75880000
-75875304
Trace of Fst1
0.6820
0.6810
Iterations
Density of Fst1
1500
500
0
0.6810
0.6815
0.6820
N = 1999 Bandwidth = 4.334e-05
Trace of Fst2
0.5195
0.5185
Iterations
Density of Fst2
0.5185
0.5190
0.5195
0.5200
N = 1999 Bandwidth = 5.148e-05
Trace of Fst3
0.1248
0.1242
Iterations
Density of Fst3
2500
1000
0.1242
0.1244
0.1246
0.1248
0.1250
0.1252
N = 1999 Bandwidth = 2.725e-05
Trace of Fst4
0.4900
0.4890
Iterations
Density of Fst4
0.4885
0.4890
0.4895
0.4900
0.4905
N = 1999 Bandwidth = 4.935e-05
Trace of Fst5
0.4925
0.4915
Iterations
Density of Fst5
0.4910
0.4915
0.4920
0.4925
N = 1999 Bandwidth = 4.796e-05

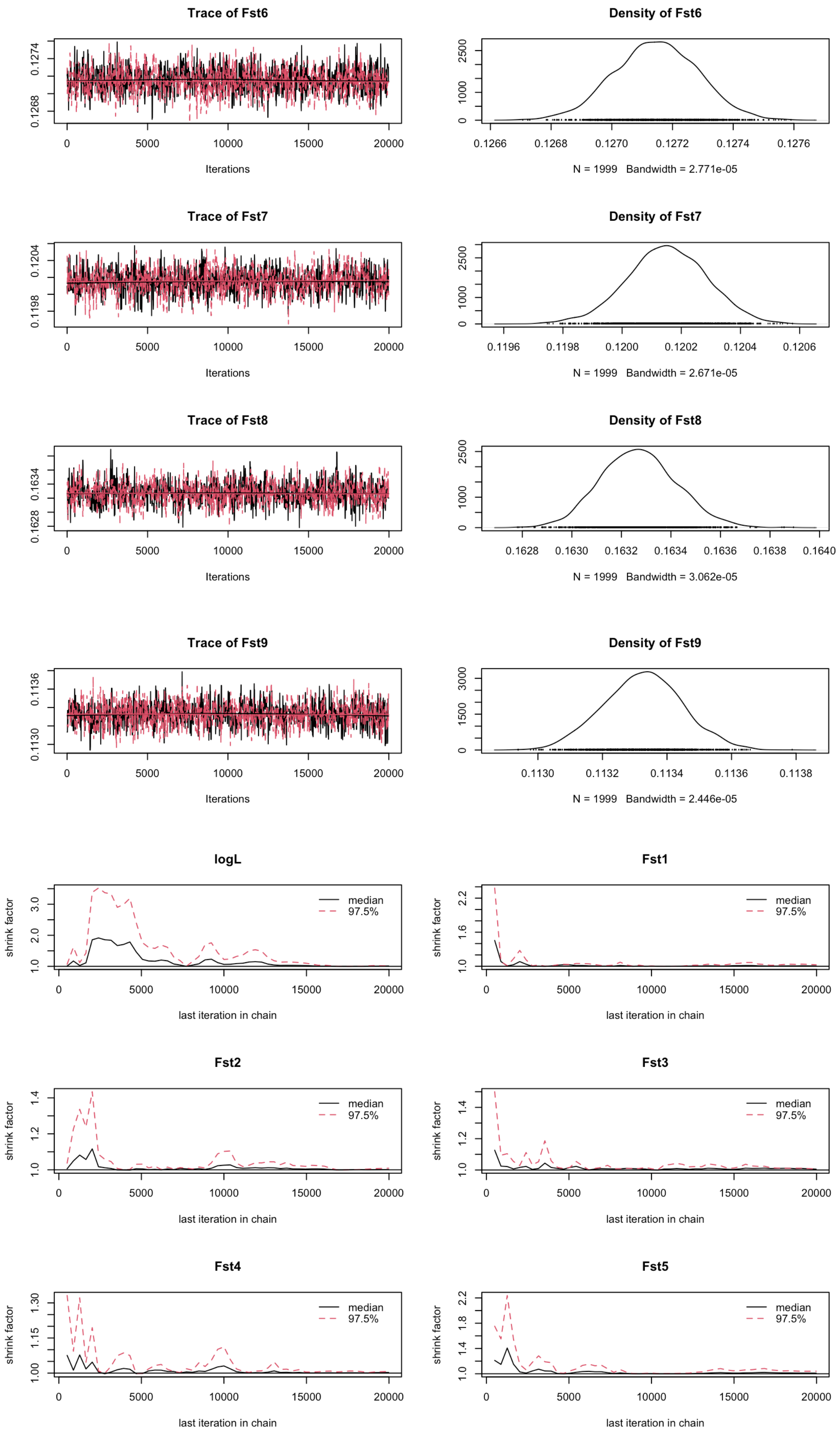

Trace of Fst6
Iterations
Density of Fst6
N = 1999 Bandwidth = 2.771e-05
Trace of Fst7
Iterations
Density of Fst7
N = 1999 Bandwidth = 2.671e-05
Trace of Fst8
Iterations
Density of Fst8
N = 1999 Bandwidth = 3.062e-05
Trace of Fst9
Iterations
Density of Fst9
N = 1999 Bandwidth = 2.446e-05
logL
Fst1
Fst2
Fst3
Fst4
Fst5
shrink factor
last iteration in chain
median
97.5%

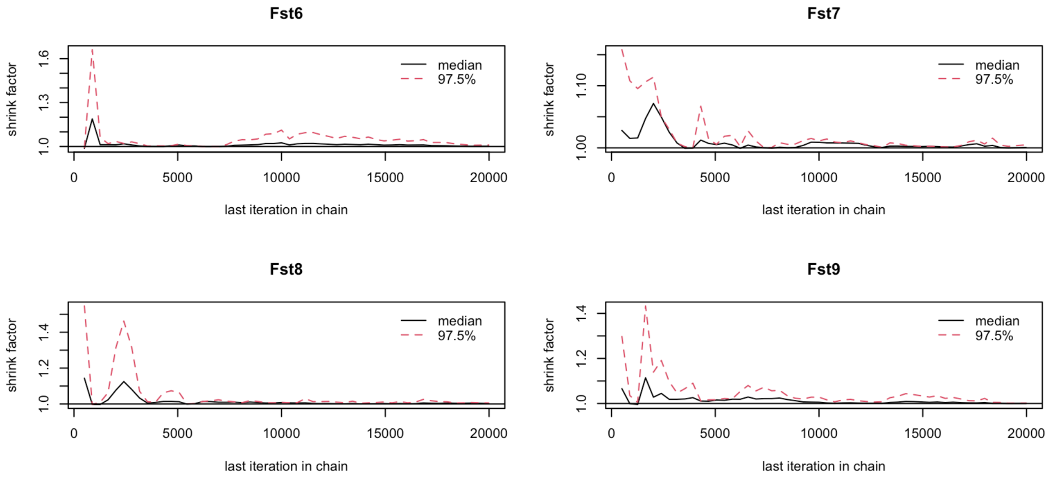

Figure S10. Trace and diagnostic Gelman plots for BayeScEnv MCMC chains with CT_{min} as the environmental variable. Trace plots show parameter values across iterations of MCMC chains. Gelman plots show the Gelman-Rubin shrink factors that summarise variation in parameter estimates across the two chains that were run.

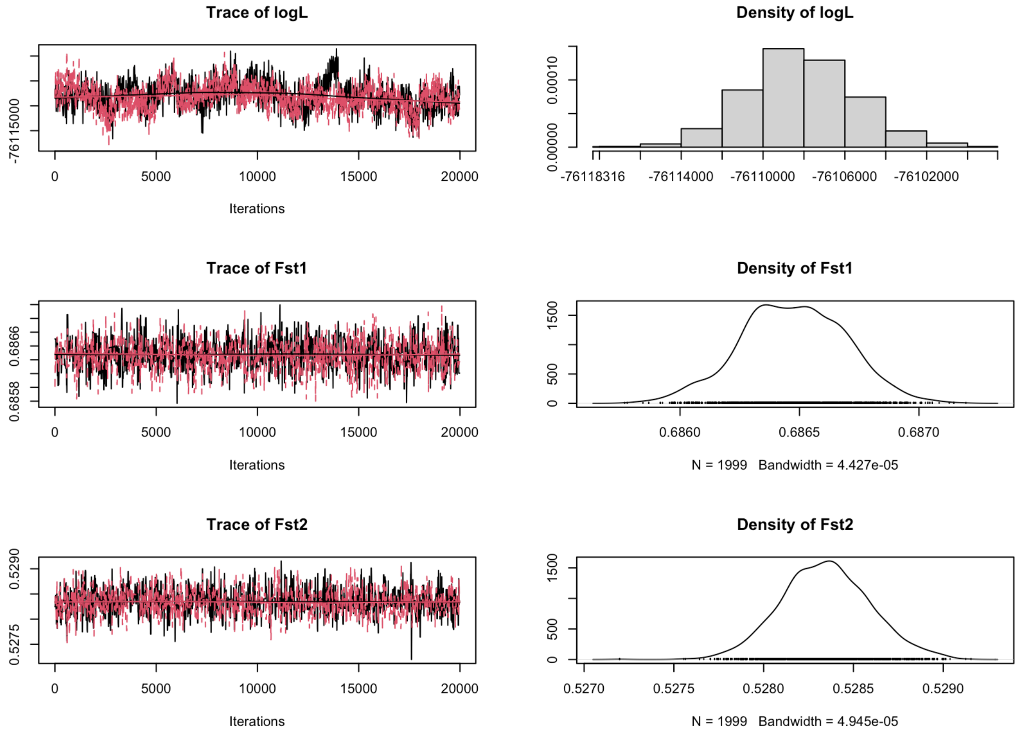

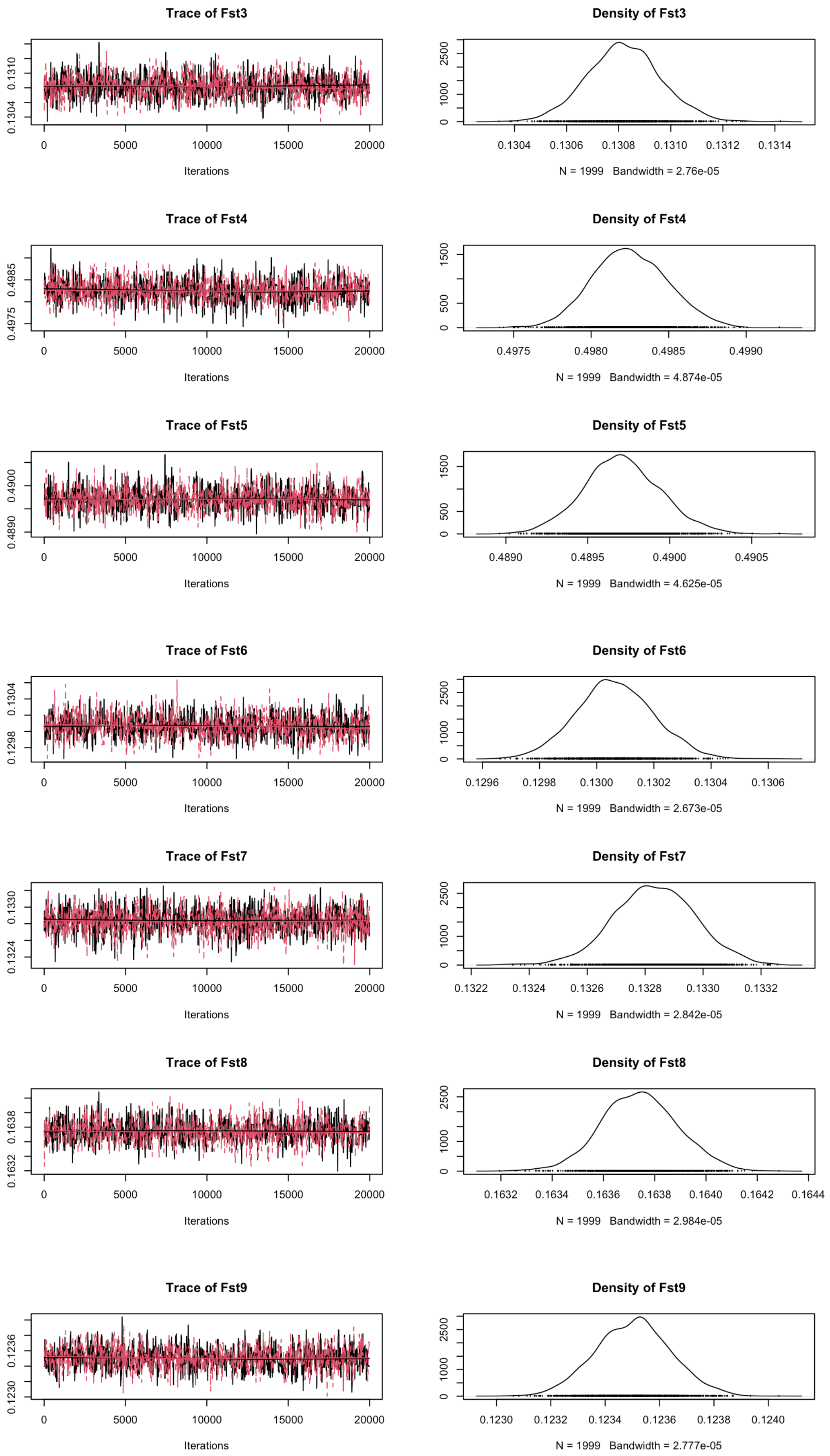
Trace of Fst3
0.1304 0.1310
0 5000 10000 15000 20000
Iterations
Density of Fst3
0 1000 2500
0.1304 0.1306 0.1308 0.1310 0.1312 0.1314
N = 1999 Bandwidth = 2.76e-05
Trace of Fst4
0.4975 0.4985
0 5000 10000 15000 20000
Iterations
Density of Fst4
0 500 1500
0.4975 0.4980 0.4985 0.4990
N = 1999 Bandwidth = 4.874e-05
Trace of Fst5
0.4890 0.4900
0 5000 10000 15000 20000
Iterations
Density of Fst5
0 500 1500
0.4890 0.4895 0.4900 0.4905
N = 1999 Bandwidth = 4.625e-05
Trace of Fst6
0.1298 0.1304
0 5000 10000 15000 20000
Iterations
Density of Fst6
0 1000 2500
0.1296 0.1298 0.1300 0.1302 0.1304 0.1306
N = 1999 Bandwidth = 2.673e-05
Trace of Fst7
0.1324 0.1330
0 5000 10000 15000 20000
Iterations
Density of Fst7
0 1000 2500
0.1322 0.1324 0.1326 0.1328 0.1330 0.1332
N = 1999 Bandwidth = 2.842e-05
Trace of Fst8
0.1632 0.1638
0 5000 10000 15000 20000
Iterations
Density of Fst8
0 1000 2500
0.1632 0.1634 0.1636 0.1638 0.1640 0.1642 0.1644
N = 1999 Bandwidth = 2.984e-05
Trace of Fst9
0.1230 0.1236
0 5000 10000 15000 20000
Iterations
Density of Fst9
0 1000 2500
0.1230 0.1232 0.1234 0.1236 0.1238 0.1240
N = 1999 Bandwidth = 2.777e-05

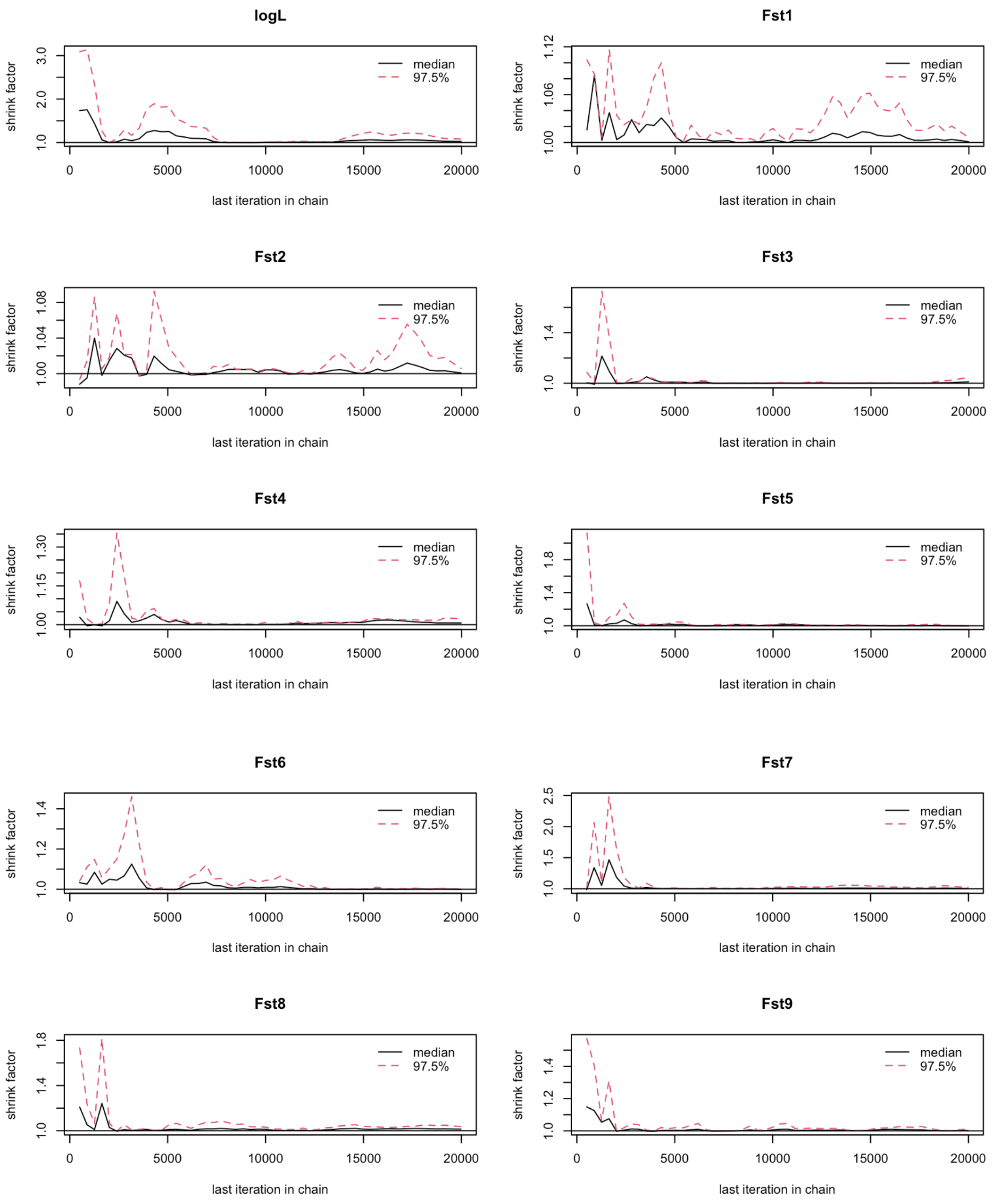

Figure S11. Trace and diagnostic Gelman plots for BayeScEnv MCMC chains with CCRT as the environmental variable. Trace plots show parameter values across iterations of MCMC chains. Gelman plots show the Gelman-Rubin shrink factors that summarise variation in parameter estimates across chains. These plots show data from two chains that were run.

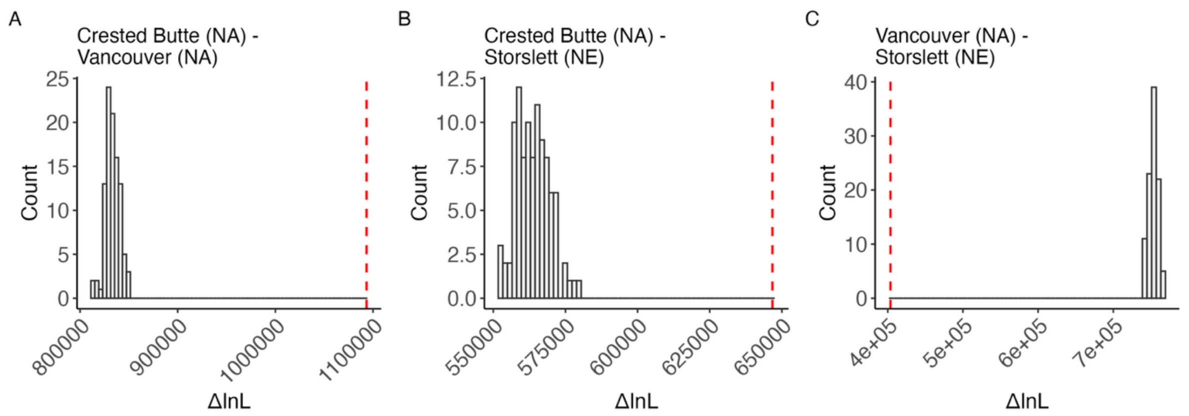

Figure S12. Model comparison was conducted for the following population pairs: (A) Crested Butte (North America, NA) and Vancouver (NA), (B) Crested Butte (NA) and Storslett (Fennoscandia, North Europe, NE), and (C) Vancouver (NA) and Storslett (NE). Datasets simulated under the strict divergence model (DIV) were fitted to both the DIV model and the best-fit isolation with migration (IM) model (see Table S19). The null distribution of ΔlnL was compared to the observed ΔlnL (indicated by vertical dashed lines). If the observed ΔlnL falls on the right side of the null distribution, it indicates that the IM model fits significantly better than the DIV model. The analysis was based on blocks of 256bp and was performed on colinear non-repetitive intergenic regions to minimize the direct effects of selection.

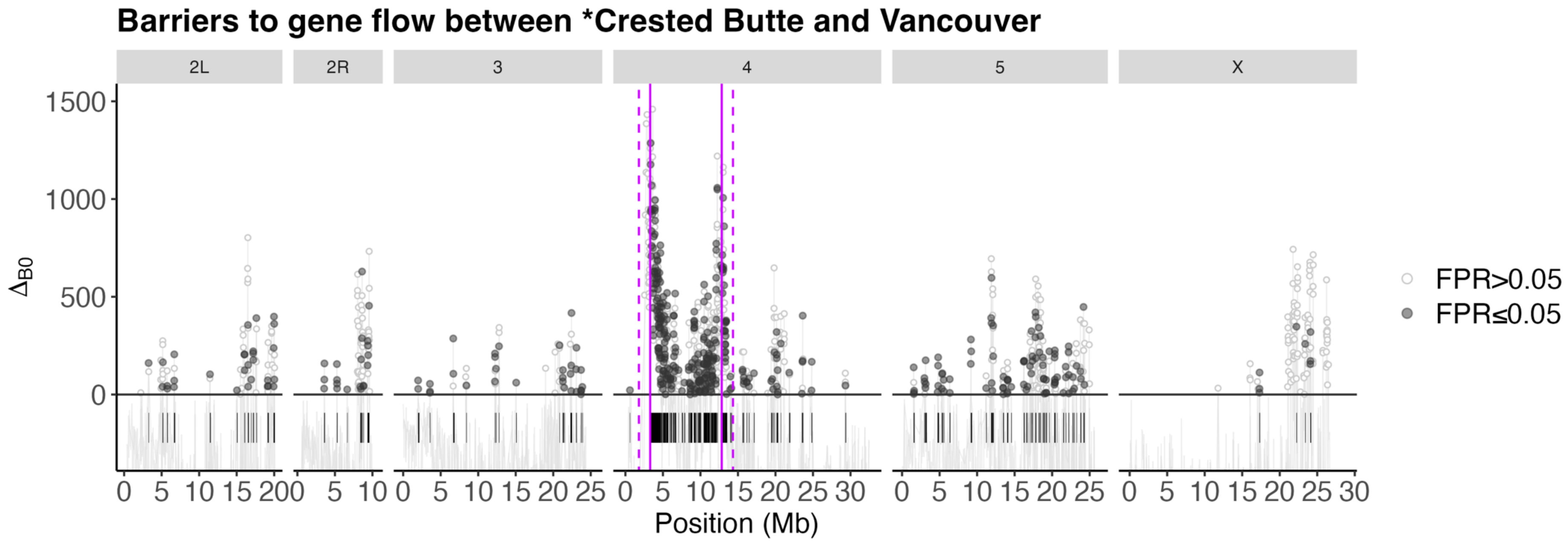

Figure S13. Support for locally reduced m_e between Crested Butte (* inversion fixed) and Vancouver (inversion absent) was measured via positive Δ_{B0} (data points above the dashed horizontal line) and a false positive rate at 5%. Barrier regions ($\Delta_{B0} > 0$ and FPR≤0.05) are marked with black segments. The solid vertical lines represent the inversion breakpoints, while the dashed vertical lines mark a conservative estimate of 1.5Mb beyond the breakpoints, where recombination is expected to remain reduced.

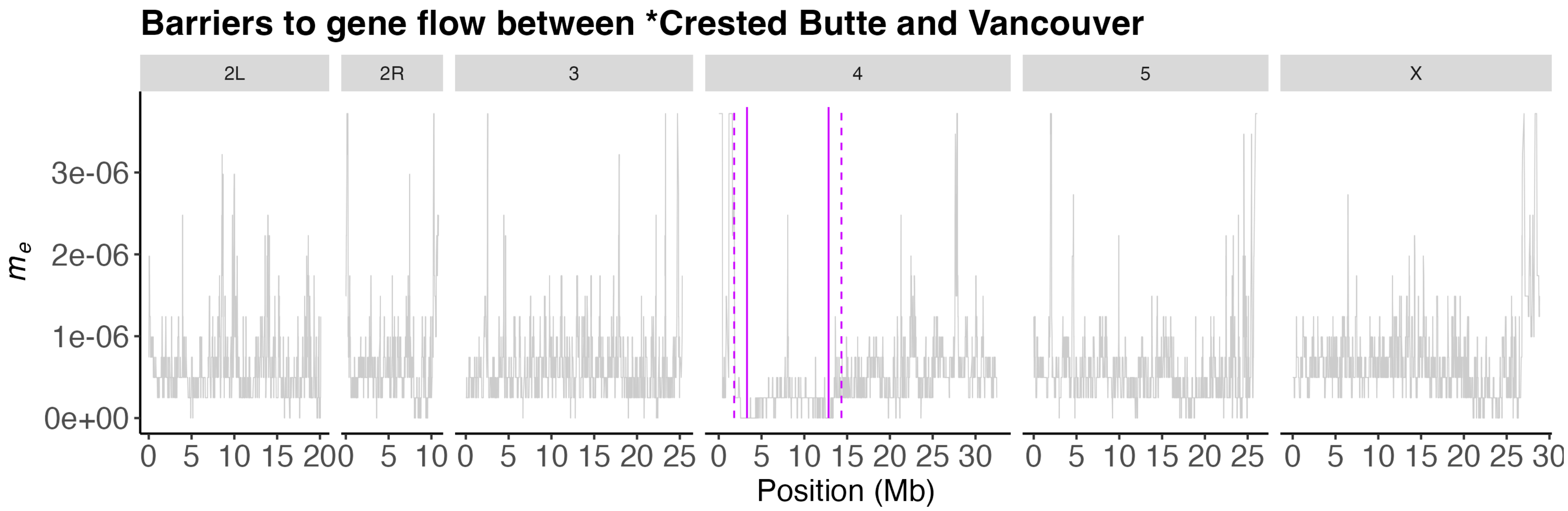

Figure S14. Window-wise variation in the effective migration rate (m_e) between Crested Butte (* inversion fixed) and Vancouver (inversion absent). The solid vertical lines represent the inversion breakpoints, while the dashed vertical lines mark a conservative estimate of 1.5Mb beyond the breakpoints, where recombination is expected to remain reduced.

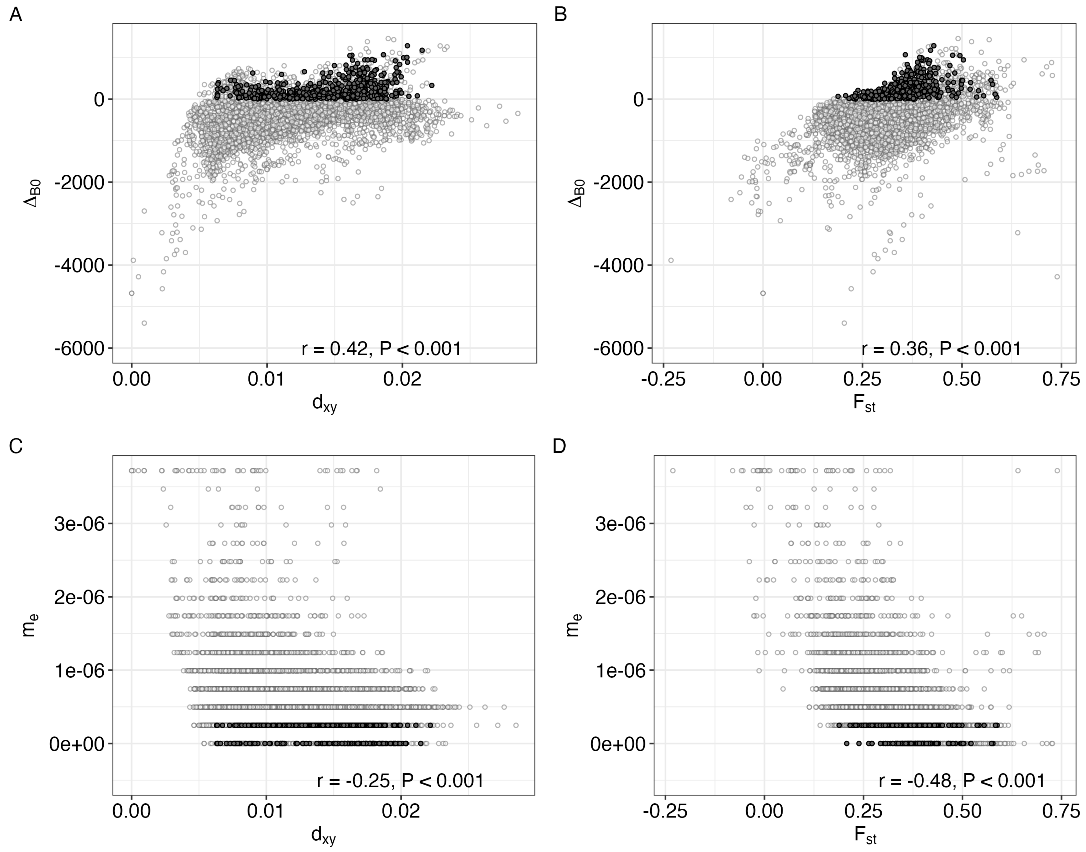

Figure S15. Pearson correlation analysis between (A) Δ_{B0} and d_{xy}, (B) Δ_{B0} and F_{st}, (C) m_e and d_{xy}, (D) m_e and F_{st}. The strongest barriers to gene flow (Δ_{B0}>0 and FPR ≤0.05, marked with closed circles), only partially overlap with d_{xy} and F_{st} outliers. m_e and d_{xy} or F_{st} are not correlated.

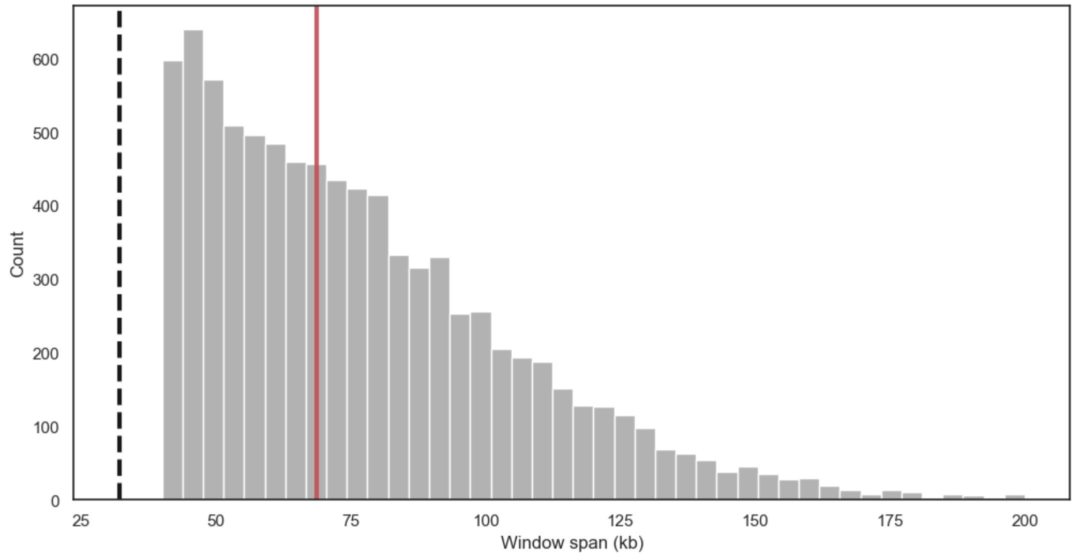

Figure S16. Window span distribution. The genome was analysed in windows of a fixed number of blocks (125 blocks, block size 256bp), which results in a minimum window span of 32 kb (dashed black line). Given that the analysis included only intergenic sequences (repetitive regions excluded), the real window span was usually greater than 32kb (median 68.6kb). Median window span is marked with a solid red line.

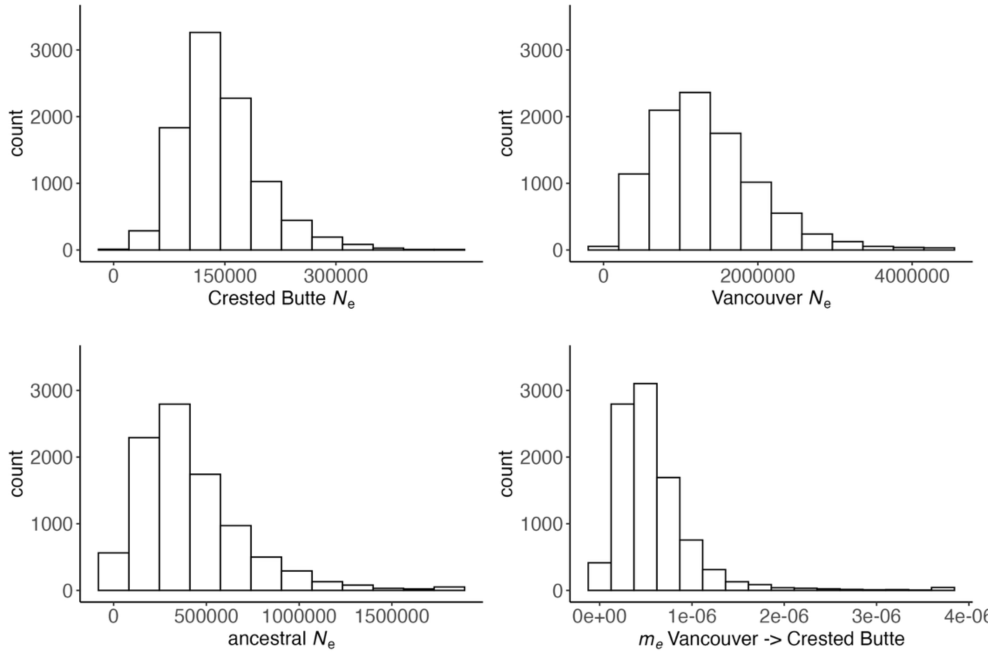

Figure S17. Variation in effective population sizes of Crested Butte (inversion fixed), Vancouver (inversion absent) and their shared ancestral population, and in the migration rate (m_e) from Vancouver to Crested Butte (forward in time) across sliding windows. The number of bins in each distribution corresponds to points in the 12 × 12 × 12 × 16 parameter grid used for inference.

Supplementary references

Fick S, Hijmans R. 2017. WorldClim 2: New 1-km spatial resolution climate surfaces for global land areas. *International Journal of Climatology* 37.

Poikela N, Laetsch DR, Hoikkala V, Lohse K, Kankare M. 2024. Chromosomal inversions and the demography of speciation in *Drosophila montana* and *Drosophila flavomontana*. *Genome Biology and Evolution* 16:evae024.

Tahami MS et al. 2024. Transposable elements in *Drosophila montana* from harsh cold environments. *Mobile DNA* 15:18.

Thorvaldsdottir H, Robinson JT, Mesirov JP. 2013. Integrative Genomics Viewer (IGV): high-performance genomics data visualization and exploration. *Briefings in Bioinformatics* 14:178–192.

Wiberg RAW, Tyukmaeva V, Hoikkala A, Ritchie MG, Kankare M. 2021. Cold adaptation drives population genomic divergence in the ecological specialist, *Drosophila montana*. *Molecular Ecology* 30:3783–3796.
